# A suitable RNA preparation methodology for whole transcriptome shotgun sequencing harvested from *Plasmodium vivax*-infected patients

**DOI:** 10.1038/s41598-021-84607-w

**Published:** 2021-03-03

**Authors:** Catarina Bourgard, Stefanie C. P. Lopes, Marcus V. G. Lacerda, Letusa Albrecht, Fabio T. M. Costa

**Affiliations:** 1 Laboratory of Tropical Diseases, Prof. Dr. Luiz Jacintho da Silva, Department of Genetics, Evolution, Microbiology and Immunology, Institute of Biology, University of Campinas—UNICAMP, Campinas, SP Brazil; 2 Instituto Leônidas & Maria Deane, Fundação Oswaldo Cruz—Fiocruz, Manaus, AM Brazil; 3 Fundação de Medicina Tropical Dr. Heitor Vieira Dourado—FMT-HVD, Gerência de Malária, Manaus, AM Brazil; 4 Instituto Carlos Chagas, Fundação Oswaldo Cruz—Fiocruz, Curitiba, PR Brazil

**Keywords:** Parasitology, Transcriptomics

## Abstract

*Plasmodium vivax* is a world-threatening human malaria parasite, whose biology remains elusive. The unavailability of in vitro culture, and the difficulties in getting a high number of pure parasites makes RNA isolation in quantity and quality a challenge. Here, a methodological outline for RNA-seq from *P. vivax* isolates with low parasitemia is presented, combining parasite maturation and enrichment with efficient RNA extraction, yielding ~ 100 pg.µL^−1^ of RNA, suitable for SMART-Seq Ultra-Low Input RNA library and Illumina sequencing. Unbiased coding transcriptome of ~ 4 M reads was achieved for four patient isolates with ~ 51% of transcripts mapped to the *P. vivax* P01 reference genome, presenting heterogeneous profiles of expression among individual isolates. Amongst the most transcribed genes in all isolates, a parasite-staged mixed repertoire of conserved parasite metabolic, membrane and exported proteins was observed. Still, a quarter of transcribed genes remain functionally uncharacterized. In parallel, a *P. falciparum* Brazilian isolate was also analyzed and 57% of its transcripts mapped against IT genome. Comparison of transcriptomes of the two species revealed a common trophozoite-staged expression profile, with several homologous genes being expressed. Collectively, these results will positively impact vivax research improving knowledge of *P. vivax* biology.

## Introduction

*Plasmodium vivax* is the most prevalent malaria parasite outside Sub-Saharan Africa, causing the most geographically widespread type of malaria, placing millions of people at risk of infection^1^. Infection occurs in genetically distinct populations with heterogeneous resistance to chloroquine, probably as a result of individual responses in a host–parasite relation^2–5^. Severe clinical complications, although scarce^6^, have been of great concern. Nevertheless, the lack of a reliable in vitro system for long term *P. vivax* culture^7,8^ restricts the study of its biology to endemic referral hospitals.

The *P. vivax* Salvador-1 (Sal-1) primate adapted strain, sequenced more than a decade ago^9^, has opened new possibilities for molecular biology studies and is still currently being used as the reference genome^10,11^. A recent publication of the *P. vivax* P01 genome with improved scaffold assembly^12^, lead to meaningful insights into *P. vivax* biology through the genomic and transcriptomic data. There are many ongoing transcriptome studies underlying several aspects of *P. vivax* pathogenesis, such as drug resistance^13,14^, disease severity^15^, the active sporozoite^16^ versus dormant liver-stage^14^ and/or other parasite blood stages^17–19^, and gametocyte differentiation^20,21^. Moreover, comparative transcriptome analysis of *P. falciparum*^22^ and *P. vivax*^19^ would be key to access the biological and clinical differences between these human malaria parasite species^8^, impacting considerably on drug and vaccine design.

The capacity to isolate, enrich and mature ex vivo* P. vivax* clinical isolates^23–25^ opened new avenues for high-throughput transcriptome sequencing analyses^19,26–28^. However, the very low parasitemias^29^ and the multi-clonality infections observed in a single patient^30^, have hindered further progress into *P. vivax* transcriptomics. The application of Whole Transcriptome Shotgun Sequencing (WTSS), also known as RNA-seq (high throughput sequencing of the transcriptome), is a powerful approach to identify strain-specific patterns of gene expression associated with parasite virulence and host–pathogen interactions^31^.

To overcome these difficulties, a methodologic framework was developed which allowed to successfully achieve all steps from RNA isolation in a set of lower parasitemia from *P. vivax* Amazonian isolates to an unbiased coverage WTSS. Also, the transcriptome analysis of a Brazilian *P. falciparum* isolate was performed. Quantity and quality of parasite RNA as well as reproducibility of RNA-seq results are discussed here. This approach enables genome-wide expression studies in *P. vivax* at endemic laboratories that will shed light on its pathogenicity, pinpoint resistance mechanisms, and facilitate validation of parasite stage-specific drug targets.

## Results

### RNA isolation methods and quality control assessment

Eight RNA isolation approaches were tested in a panel of *P. falciparum* samples with a wide-ranging parasite density (10^2^ to 10^7^ per sample). Protocols from single-step RNA isolation by acid guanidinium thiocyanate–phenol–chloroform extraction and a TRIzol based, to user-friendly kits were evaluated (Supplementary Fig. S1). Sizing, quantity, and quality of RNA was estimated by Bioanalyzer platform (Supplementary Fig. S2). The TRIzol extraction and the RNeasy Micro kit (preserved in RNAlater) were the two methods yielding samples with the highest total RNA concentrations showing the more reliable RNA Integrity Number (RIN) values. To evaluate the limitations of downstream reactions three housekeeping genes were amplified (*DNA repair helicase*, *gamma GCS* and *seryl-tRNA ligase*) by qRT-PCR. As expected, an inverse relation between quantities of parasites and the amount of total RNA extracted versus the mean Cq value was observed. This was consistent for all extraction methods (Supplementary Fig. S3, S4 and S5). RNA samples extracted using manual protocols, although initially presenting higher quantifications, such as those extracted with TRIzol, often lead to samples with high Cq signals, crossing the reaction detection limits (RT-corresponding controls) (Supplementary Fig. S3). Surprisingly, similar result was observed for the RNeasy Plus Micro Kit, with or without preservation in RNAlater (Supplementary Fig. S4). In addition, samples extracted with methods using TRIzol had lower amplification efficiency (Supplementary Fig. S5). RNA samples extracted using RNeasy Micro kit and Direct-zol RNA MiniPrep kits from previously preserved biological material, showed lower Cq signals, within the limits of detection, especially in samples with 10^3^ to 10^5^ Pf-iE (Supplementary Fig. S4), amounts expected to be found on *P. vivax* isolates.

**Figure 1:**
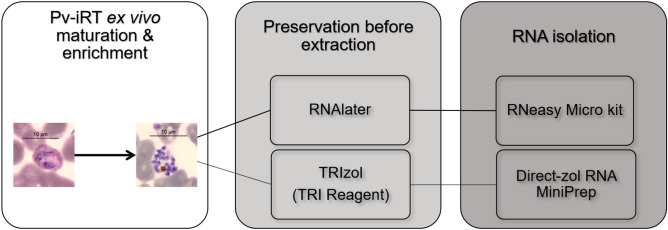
*P. vivax* isolation, short-term ex vivo maturation, enrichment and total RNA extraction. Blood samples were immediately processed. White blood cells were depleted by CF11 filtration^25^. The early blood staged parasites were short-term cultured ex vivo to allow maturation followed by Percoll gradient enrichment^23^. Total number of Pv-iRTs was accessed before preservation in RNAlater or TRI Reagent at − 80 °C. RNA extraction was executed using the RNeasy Micro kit.

**Figure 2:**
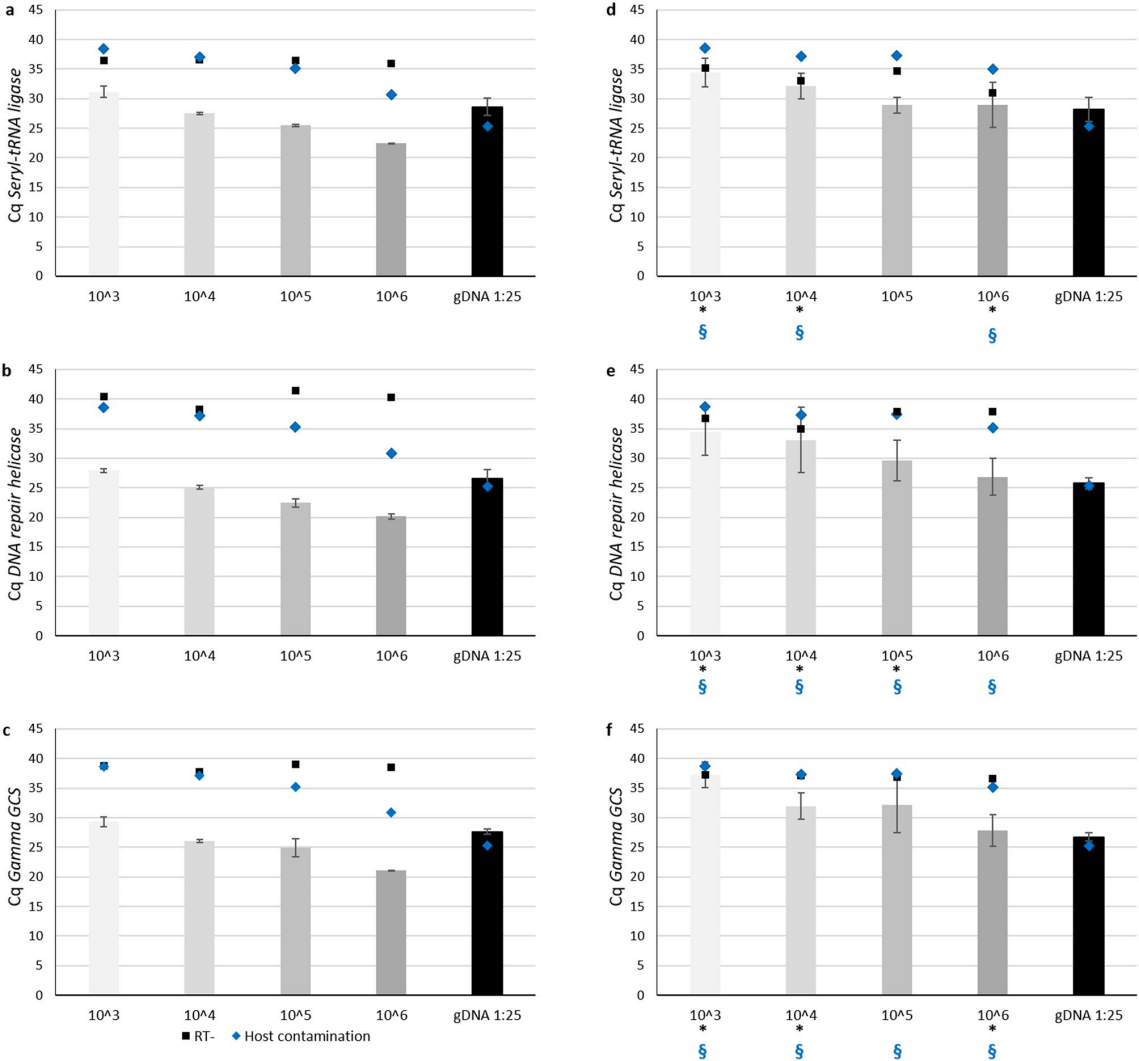
qRT-PCR of *P. vivax* total RNA samples. qRT-PCR amplification of housekeeping genes *seryl tRNA synthetase* (**a**,**d**), *DNA repair helicase* (**b**,**e**) and *gamma-glutamylcysteine synthetase* (**c**,**f**)*,* from the RNA samples of one Pv-iRT field-isolate (10^3^ in light gray to 10^6^ in dark gray), preserved in RNAlater or TRI Reagent and extracted by RNeasy Micro kit (**a**–**c**) or Direct-zol RNA MiniPrep (**d**–**f**), respectively. Bars and error bars represent the mean Cq and standard deviations (SD), respectively. Black square marks represent average Cq of RT- reactions, when amplification was observed before the last (45th) cycle stage. Blue diamond marks represent mean Cq amplification of human TLR9 gene for host contamination detection. Black * indicate when the mean Cq for each sample is higher than the limit of detection (3 times the Cq SDs from the correspondent RT- mean Cq) and blue § indicate when the mean Cq is higher than the mean Cq for host contamination detection (3 times the Cq SDs from the correspondent TLR9 mean Cq).

The two most reliable combinations of preservation and extraction protocols, RNAlater/RNeasy Micro kit and TRI Reagent/Direct-zol RNA MiniPrep (Supplementary Figs. S2–S5), were chosen to extract RNA from *P. vivax* isolates (Fig. 1). After isolation, enrichment, and short-term ex vivo maturation, RNA from a set of samples with different number of parasites (10^3^ to 10^6^) was extracted. Bioanalyzer quality controls are shown in Supplementary Figure S6. The obtained RNA concentrations were well above the 50 pg.µL^−1^ for the Agilent kit used. But the RIN values range observed for *P. vivax* RNAs were comparable to those of *P. falciparum* (Supplementary Fig. S2), but higher for the samples extracted using the RNAlater/RNeasy Micro kit. The RNA extracted from these isolates was then amplified by qRT-PCR for the three housekeeping genes as previous (Fig. 2). It was observed that, although the results were similar for both conditions, Cq values were higher when the sample was preserved in TRI Reagent and then extracted using the Direct-zol RNA MiniPrep (Fig. 2 and Supplementary Fig. S7), often crossing the correspondent RT- signals and dangerously overlaping the host contamination signal (corresponding TLR9 Cq). In *P. vivax* samples extracted using the RNAlater/RNeasy Micro kit, the gDNA contamination from the parasite was limited, seen on the more consistent separation between the Cq of the three housekeeper genes against the corresponding RT- controls, and host contamination was reduced and not interfering in the qRT-PCR reactions (Supplementary Fig. S7). Combining those results, the RNeasy Micro kit was the most reliable option.

### P. vivax low input cDNA synthesis, library preparation and sequencing

RNA extractions of 7 different samples from 4 different *P. vivax*-infected reticulocyte (Pv-iRT) isolates (62U15, 66U15, 93U15 and 101U15) were performed using the RNeasy Micro kit and their quantity and quality evaluated by Bioanalyzer platform (Supplementary Fig. S8). To set guide thresholds for the minimal input requirements for RNA-seq, a control sample was included, with approximately 10^6^ parasites trophozoite staged of *P. falciparum* S20 strain^32^. On average 101.5 pg.µL^−1^ of RNA, ranging from ~ 10.5 to ~ 522 pg.µL^−1^, was obtained (Supplementary Table S2). For *P. falciparum* S20 strain, the lowest amount of total RNA (~ 1.2 pg.µL^−1^) was acquired. Following previous observations, RIN score often fail due to low quantities of RNA (Supplementary Table S2 and Fig. S8). Given the low amounts of *P. vivax* total RNA (~ 100 to 1000 pg per sample; Supplementary Fig. S9a), SMART-seq technology was chosen. As expected, the amount of cDNA obtained correlates with the initial amount of RNA used (Supplementary Fig. S9b; Supplementary Table S3) and the sequencing-ready library for the Illumina platform (Supplementary Table S4).

### Whole transcriptome shotgun sequencing data analysis

A total of 4,004,593 raw reads were obtained, with on average of 451,094 paired end reads (100 bp) per sample (Supplementary Table S5). The *P. vivax* reads had a mean of 47% GC content, while *P. falciparum* had 30%. FastQC revealed good sequence quality and trimming steps only excluded a minor fraction of reads (≤ 4%) (Supplementary Table S5). The remaining reads were aligned and mapped to the *P. vivax* P01 and *P. falciparum* IT genomes, respectively (Supplementary Table S6). Samples with similar number of parasites had similar initial amounts of raw reads and similar level of read mapping output (Supplementary Fig. S9c). When the number of Pv-iRT harvested dropped to ≤ 10^4^, there was a significant reduction in the amount of RNA and a significant drop in the number of output reads, which reflects a lower rate of concordant pair aligned reads (Supplementary Table S6). Altogether, these five samples (93U15_21, 93U15_22, 101U15_20, 101U15_23 and 101U15_24) had a considerable reduction of the total number of aligned and mapped reads (Supplementary Tables S5 and S6). Sequences showing multiple or discordant alignments were excluded from further downstream analysis. All aligned and mapped paired reads were subsequently assigned to features, *i.e.* genes (Supplementary Table S8). The number of mapped reads varied among 93U15 and 101U15 technical replicates with exons detected in common, as well as many detected only one of the replicates. The agreement (Kappa coefficient) between these technical replicates within an isolate ranges from 0.304 to 0.526 and among the two isolates discrepancies were larger (ranging from 0.037 to 0.446), indicating that replicates with higher coverage (101U15_23 and 24) presented a better agreement (Supplementary Table S10). Isolates 62U15, 66U15 and 101U15_23 presented the highest agreement in between, while the lowest agreements were observed in comparisons involving 93U15_21, the isolate with lowest initial quantity of Pv-iRTs, thus less number of mapped reads (Supplementary Table S10 and Fig. S9).

Given the characteristics of the field samples, the human host RNAs were also investigated, by aligning and mapping the reads that did not align to the *P. vivax* P01 reference genome. Less than 20% of the reads were mapping to the human genome, with a wide range of concordant pair alignment rates between the different samples, from as little as 0.3% to 14.7% (Supplementary Tables S7 and S9).

### Plasmodium spp. and human expression profiles

**Figure 3:**
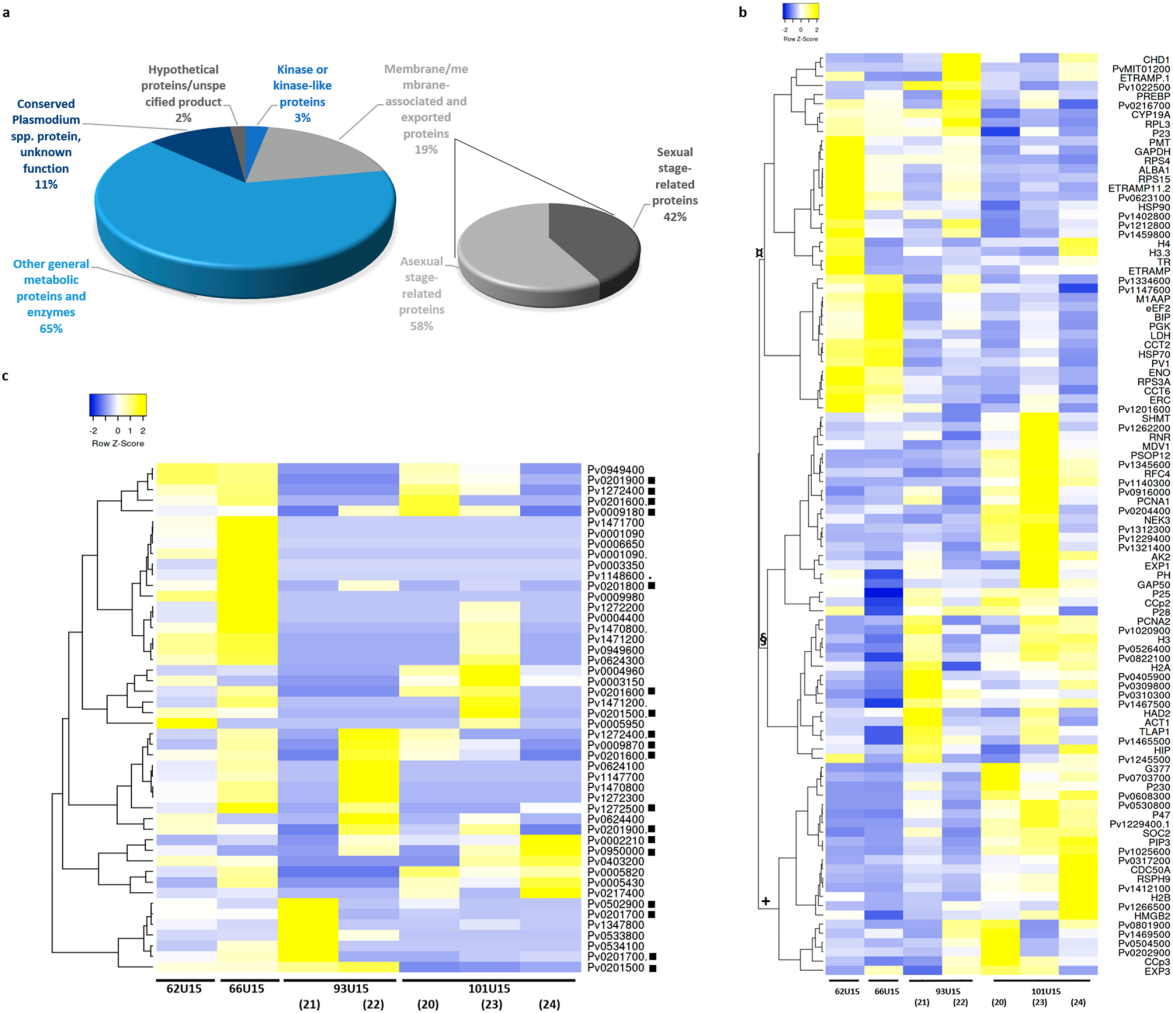
Most expressed *P. vivax* genes. (**a**) Pie charts showing the top 100 most expressed genes from RNA sequencing of *P. vivax* field isolates, grouped by general *Plasmodium* spp**.** metabolic proteins and enzymes (65%, light blue), membrane, membrane-associated and exported proteins (19%, light grey), conserved Plasmodium-like proteins of unknown function (11%, dark blue), kinases or kinase-like proteins (3%, blue) and hypothetical/unspecified product proteins (2%, grey). The amplified right-hand side pie chart shows the percentage of asexual (58%, light grey) and asexual (42%, dark grey) membrane, membrane-associated and exported proteins (19%, light grey). (**b**) Heatmap clustering of top 100 most expressed *P. vivax* exons from the RNA sequenced filed isolates using complete linkage hierarchical clustering method and Pearson’s distance measurement method for computing distance between rows and columns^75^. The three main branches are indicated by symbols + , § and ¤. (**c**) Heatmap clustering of top 48 most expressed transcripts of *P. vivax* PIR genes from the RNA sequenced filed isolates, using complete linkage hierarchical clustering method and Pearson’s distance measurement method for computing distance between rows and columns^75^. Twenty-one PIR genes are expressed in all isolates (black dots).

**Figure 4:**
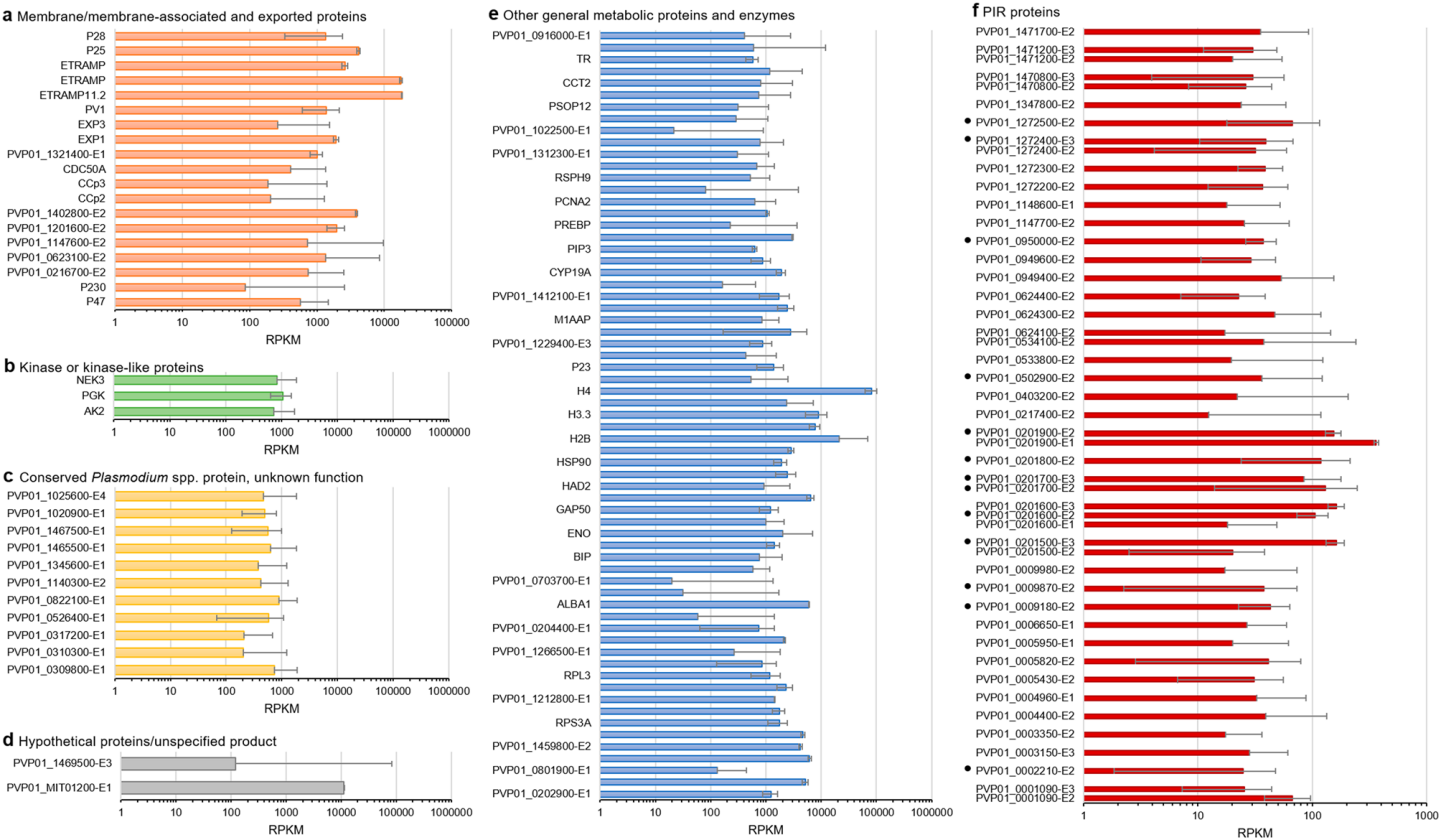
Expression levels of *P. vivax* transcripts. Expression levels in gene coverage within the four different *P. vivax* isolates of (**a**) membrane and membrane-associated and exported proteins, (**b**) kinase or kinase-like proteins, (**c**) conserved *Plasmodium* spp. proteins of unknown function, (**d**) hypothetical proteins and unspecific products, (**e**) other general metabolic proteins and enzymes, and (**f**) PIR proteins. Black dots highlight the *pir* transcripts expressed across all isolates. Error bars represent the standard deviation between exons RPKM across all samples (Supplementary Table S11).

There was a great overlap between the most expressed genes in each isolate, of which round 13% of expressed *P. vivax* proteins identified have not been characterized yet (Fig. 3a). However, a substantial heterogeneity was observed for genes related to membrane and exported proteins (Fig. 3b). A careful look into the most expressed genes showed that they clustered in three major branches (Fig. 3b, identified by + , § and ¤). As for *P. falciparum,* the 100 most expressed *P. vivax* genes in all isolates were further classified in five different main groups: (1) *Plasmodium* spp. metabolic proteins and enzymes (65%); (2) membrane, membrane-associated and exported proteins (19%); (3) conserved *Plasmodium*-like proteins of unknown function (11%); (4) kinases or kinase-like proteins (3%) and (5) hypothetical/unspecified product proteins (2%) (Figs. 3a, 4a–e, Supplementary Figs. S10, S11 and Tables S12 and S13). One of the most interesting, yet poorly characterized gene families is the *Plasmodium* interspersed repeat multigene family (PIR)^33,34^. Although these genes were found to be not highly expressed, 39 *pir* genes were identified (≥ 10 reads per transcript) to 48 different exons in these *P. vivax* isolates (Fig. 3c). Heatmap clustering shows different patterns of expression of these genes for each isolate (Fig. 3c and Supplementary Table S11), with variable expression levels (Fig. 4f).

As anticipated, one detected highly expressed *P. falciparum* membrane protein was the knob-associated histidine-rich protein (KAHRP) specie specific, otherwise families of *Plasmodium* spp. membrane proteins such as ETRAMP, mature parasite-infected erythrocyte surface antigen (MESA), merozoite surface proteins (MSP) and EXP were identified (Supplementary Fig. S11), as previously reported^35,36^.

Given the importance of understanding the human expression profiles during vivax malaria, the top 100 most expressed human genes that could be identified from this RNA-seq data were also analyzed. Several immune related genes were identified, including those associated with the human host phagocytosis, neutrophil-related and other pathways (Supplementary Fig. S12e and Table S14).

## Discussion

*P. vivax* infections are characterized by one order of magnitude lower parasitemia than *P. falciparum*. With low number of parasites and parasitic RNA being a small fraction compared to a mammalian cell^37^, a minor amount of parasite RNA is expected. The sequenced RNA from the parasite often sum up to less than half of the total raw data^13,20,26^. Therefore, there is a need to optimize all process to get the best quality of parasite RNA for gene expression studies. Here, a detail method is described to establish a *P. vivax* RNA-seq approach.

Several methods of sample preservation combined with RNA isolation in a panel of *P. falciparum* samples that mimic the low parasitemia verified in *P. vivax* infected patients were tested (Supplementary Fig. S1) and further validated using *P. vivax* isolates (Fig. 1). The Bioanalyzer was used to evaluate the RNA quantity, quality, and purity, where TRIzol based protocols (both manual and kit) yielded higher quantities of RNA (Supplementary Fig. S2). Even if resulting in samples with higher RNA concentrations, manual protocols are much more laborious and time-consuming when compared with kits. Most importantly, often leave traces of solvent contaminants (TRIzol, ethanol, etc.), later seen on less efficient qPCR reactions (Supplementary Fig. S3 and S5). Surprisingly, the RNeasy Plus Micro kit, in all similar to RNeasy Micro kit exception made for a step where a gDNA eliminator columns is used to avoid additional DNase treatment, showed too high qPCR efficiencies characteristic of samples with template (parasite gDNA) contaminations (Supplementary Fig. S4 and S5). By qRT-PCR, it was possible to amplify *Plasmodium* spp. constitutively expressed genes from the extracted RNA of an initial low number of Pv-iRT (10^3^ parasites per sample; Fig. 2), where we were more successful when using RNAlater/RNeasy Micro kit method. However, given the availability of liquid nitrogen and for all the samples destined to RNA sequencing, we opted by flash freezing the isolates, instead of freezing them at − 80 or − 20 °C using RNAlater. The amplification of genomic DNA from *P. vivax* was reduced and did not interfere with downstream analysis (Supplementary Fig. S7). While leukocytes are completely absent from *P. falciparum *in vitro culture, CF11 filtration efficiently removed them from *P. vivax* samples^23^, as reflected a little amplification of host gene was observed (Fig. 2).

Contrary to microarray, where exploration is limited to the current tagged transcriptome, the Smart-seq technology, originally developed for single cell RNA-seq (scRNA-seq)^19,38–40^, was used for sequencing the entire transcriptome. Recently, scRNA-seq of *P. falciparum* and *P. berghei* was performed using this technology^41,42^ and it was also used to determine the transcriptional profile of *P. vivax*^18,43,44^. Its advantage for low input samples is to avoid the ribosomal RNA depletion, still ensuring that the final cDNA libraries contain the 5′ end of the mRNA and maintain a true representation of the original mRNA transcripts, by avoiding excessive rounds of PCR preamplification of the cDNA^43^. Overall, a direct relationship is observed from the initial number of parasites to the final expected sequencing output of read pairs counted per gene (Supplementary Fig. S9). Here, from 10^3^ to10^6^ parasite.µL^−1^ (Supplementary Fig. S9) using as little as ~ 10 pg.µL^−1^ of RNA (Supplementary Table S2), it was possible to sequence and obtain high quality raw reads (Supplementary Table S5), of which ~ 50% align and map to the *P. vivax* P01 (Supplementary Table S6). Note that, contrary to 62U15 and 66U15, the isolates 93U15 and 101U15 were initially unevenly subdivided into two and three samples respectively, allowing a better observation for technical limitations. The number of mapped reads varied among 93U15 and 101U15 technical replicates, where those with higher coverage presented a better agreement (101U15_23 and 24), a sign of increased consistency of exons detection across technical replicates (Supplementary Table S10). As expected, discrepancies were higher between different isolates (Supplementary Table S10)^45,46^. Isolates where the low initial quantity of Pv-iRTs (93U15_21), thus less number of mapped reads to an exon (Supplementary Fig. S9 and Table S10), have this limitation reflected in the lower agreement (Kappa coefficient) within the experiment, which must be taken into consideration. The transcripts from the four different isolates here analyzed were aligned and mapped against the *P. vivax* P01 and *H. sapiens* GRCh38 reference genomes (Supplementary Tables S6 and S7). The assembled *P. vivax* P01 reference genome^12^ was chosen, given that it represents a 10% larger assembled genome with a deeper coverage and 22% additional gene characterization relative to *P. vivax* Salvador I^9^.

Another challenge found in studying *P. vivax* expression profile is the asynchrony of parasites in patients blood, with mature parasite less present in circulating blood compared to younger stages^47^. However, later *P. vivax* stages are more abundant in transcripts that tend to outcompete other transcripts by the RNA-seq. This creates a bias towards mature parasite overrepresentation when characterizing *P. vivax* transcriptomes from blood-draw samples without undergoing further sychonization for a parasitic stage^20,25,26^. It is important to note that there is a considerable variation and degree of expression between the isolates (Fig. 4b,c). This could be explained by the quantity of starting template, the amount of host RNA and the mixes of different proportions of the several parasite stages in each isolate. However, more recent publications have reported on a significantly increased heterogeneity of expression of genes in synchronized and staged *P. vivax* transcriptomes from field isolates^46^. These factors must be taken into account when comparing data across several biological replicates.

Most of the transcripts sequenced here have been previously characterized as expressed in trophozoite stages, from throughout the *P. vivax* intraerythrocytic development cycle (IDC) (Supplementary Fig. S11)^18,20,25–28,46^. As recently reported^46^, most of the *P.* vivax expressed genes with the highest abundance were found to overlap between isolates and could be grouped by predicted protein functions (Fig. 3a). A group containing membrane, membrane-associated and exported proteins, included genes expressed during the asexual stage, such as early transcribed membrane proteins (ETRAMP), exported proteins (EXP) and parasitophorous vacuolar proteins (PV). A transcriptional characterization of these membrane, membrane-associated and exported proteins can be paramount for vaccine development. An equal representation of membrane proteins characterized as asexual or sexual parasite stages, such as the ookinete surface proteins P25 and P28 and LCCL domain-containing proteins (CCp) was observed (Fig. 4a). Given the asynchronous characteristics of these isolates and the fact that *P. vivax* not only mature from rings into schizonts, but also can develop into gametocytes^18^, the presence of a fraction of gametocytes was expected. This RNA-seq approach was sensitive enough to detect gametocyte gene expression from a sample where most parasites were trophozoites. The most highly expressed genes included P25, previously characterized as a *P. vivax* gametocyte marker, and other characteristically sexual stage expressed genes such as P28, P47, PSOP12, CCp3, PVP01_0526400 and PVP01_1345600^18,27,48–50^ (Fig. 4a).

A more detailed look was given at the 39 different *pir* genes expressed (Figs. 3c and 4f). These genes are not clonally expressed and believed to play an important role of the parasite immunovariation capacity^33^. Twenty-one exons (out of 48) belonging to PIR family were expressed in all *P. vivax* isolates (Fig. 4f). This results are in line with what has been described as immunovariant and non-clonal expression through distinct parasite populations^33^. This variation between isolates for the top most expressed *pir* genes^51^ suggests that these multigene families might be under epigenetic regulation^52^, as reported for multiple *P. falciparum* gene families^53^. Through all isolates sequenced here, overlap could be observed for the most expressed genes. Further studies should focus on analyzing the variable genes (such as *pir*) between patient isolates, since transcriptional heterogenicity could reveal a differentiated host immune response and/or a distinct parasite invasion mechanisms^15,46,51,54,55^. Furthermore, transcriptional variability of multigene families was observed towards different vivax malaria endemic regions, where there is not an overlap between the highest expressed *pir* genes reported^20,46^.

Among the most expressed genes, three kinases were found, adenylate kinase 2 (AK2), NIMA related kinase 3 (NEK3) and phosphoglycerate kinase (PGK) (Fig. 4b). An understanding of the expression dynamics of kinases through different *P. vivax* populations opens avenues in the field of drug discovery^56,57^. As expected, most of the identified genes code for *Plasmodium* spp**.** metabolic proteins and enzymes, most likely essential genes, constitutively expressed through all parasite IDC (Fig. 4d). Multiple proteins emerged, including those involved in several basal metabolic pathways, such as glycolysis, Calvin-Benson-Bassham cycle and deoxyribonucleotide de novo biosynthesis, characteristic of mature parasites stages^18,46^. Still, within these 100 most expressed *P. vivax* genes, almost a quarter of highly expressed transcripts encode conserved *Plasmodium*-like and hypothetical proteins of unknown function, yet to be functionally characterized (Figs. 3a, 4c,d). This is a lower percentage when compared to the overall functional annotation of *P. vivax* P01 reference genome^26^, where ~ 30% protein coding genes remain uncharacterized.

The expression profile of *P. falciparum* S20 was also analysed and were similar to those previously reported^35,36^. Most interestingly, many of the *P. falciparum* highest expressed gene fall into the same function categorized groups described for *P. vivax* isolates (Supplementary Figs. S10 and S11). However, there was an increase in the number of identified genes coding for *Plasmodium* spp. metabolic proteins and enzymes and a significant decrease of functionally uncharacterized ones (Supplementary Fig. S10). This might reflect the different annotation level of *P. vivax* P01 versus *P. falciparum* IT reference genomes. Many of those sequences had identified *P. vivax* homologs, also highly expressed in all isolates. Out of the 100 most transcribed *P. falciparum* S20 genes, 9% are identified as coding membrane-associated and exported proteins, as expected from a trophozoite-enriched sample.

Given the importance of better understanding the vivax malaria host–pathogen interaction, the human expression profiles were accessed here, based on the limited data available. Less than 20% of the reads were mapping to the human genome, which supports the reliability of the methodology which focuses on sequencing the *P. vivax* transcriptome. Nevertheless, a wide range of concordant pair alignment rates were observed between the different samples. As expected, several immune related genes were identified, including those associated with the human host phagocytosis, neutrophil-related and other pathways (Fig. 4f). This falls in line with the several *Plasmodium* spp. highly expressed genes implicated in host-parasite interactions, like those encoding membrane, membrane-associated or exported proteins (Fig. 4a), those involved in translation and in active parasite replication (Fig. 4e).

## Conclusion

Coupling a column-based RNA extraction kit for samples having limited number of parasites with Smart-seq cDNA libraries amplification, can help researchers to overcome one barrier to genome-wide functional analyses in *P. vivax*. This could be achieved by promoting the generation of expression data from world parasite populations within their hosts, as well as, improve the success rate of WTSS on clinical isolates, even with the typically scant resources of laboratories in malaria endemic areas.

## Methods

### Ethical approval

Informed consent was signed by all patients. All procedures, including protocols and consent forms, were approved by the Ethics Review Board of FMT-HVD (processes CAAE-0044.0.114.000-11 and 54234216.0000.0005). All methods were performed in accordance with the relevant guidelines and regulations.

### Study area, subjects and sample collection

Patient recruitment was implemented at FMT-FVD, a tertiary care center for infectious diseases in Manaus, Amazonas State, Brazil. Only adult patients were recruited. Exclusion criteria comprised severe malaria, patients under anti-malarial treatment, with *P. falciparum* malaria and/or *P. falciparum* and *P. vivax* mixed infections and pregnant women (Supplementary Table S1). After conventional thick-smear microscopic diagnosis of *P. vivax* malaria, determination of parasitaemia and before treatment was initiated, up to 8 mL of peripheral blood was collected in citrate-coated Vacutainer tubes (Becton–Dickinson). Subsequently, *P. vivax* mono-infection was confirmed by PCR analysis, as described elsewhere^58^.

### *P. vivax * isolation, enrichment and ex vivo maturation

All samples were immediately processed to obtain enriched *P. vivax* infected erythrocytes (Pv-iRTs). Plasma and buffy coat layer were removed after separation by centrifugation at 400 × *g* for 5 min at room temperature. The pellet was resuspended in an equal volume of McCoy-5A medium (Gibco) and then CF11 column filtration (Sigma) was performed to deplete white blood cells (WBC)^23,25,59^. Thin blood smears were prepared and stained with *Panótico Rápido* (Laborclin) kit, before, during and after short ex vivo culture to control the parasite maturation. Depending on the stage of parasite maturation, the early blood staged parasites were cultured to a 5% hematocrit in McCoy-5A medium supplemented with 20% of human AB serum, incubated at 37 °C with a gas mixture containing 5% CO_2_, 5% O_2_, 90% N_2_ for 18–22 h to allow them to mature to trophozoites^23,60–62^. Subsequently, completed parasite enrichment was achieved by Percoll 45% gradient as previously described^63^.

### *P. falciparum *in vitro cultures

*P. falciparum* S20^32^ was cultured according to standard procedures previously described^62^. In brief, *P. falciparum* S20 strain was cultured in purified erythrocytes from O^+^ healthy local donors in RPMI 1640 (Gibco) supplemented with 5% AlbuMAX (Gibco), sodium bicarbonate (25 mM; Sigma), hypoxanthine (100 µM; Sigma) and gentamycin (50 µg.L^−1^; Gibco). *Pf-iEs* were maintained at 2% hematocrit and incubated at 37 °C with a gas mixture containing 5% CO_2_, 5% O_2_, 90% N_2_. Cultures were synchronized with a 5% sorbitol solution and further enriched for mature stages in 60% Percoll. Thin blood smears were prepared and stained with *Panótico Rápido* (Laborclin) kit during in vivo culture to control the parasite maturation and parasitemia.

### RNA extraction and quality control

*P. falciparum* total RNA was extracted from a set of cultures with 10^2^ to 10^7^ Pf-iEs (in triplicate) using in total five different methods: two different manual protocols and three different commercial kits were used (Supplementary Fig. S1). The single-step method of RNA isolation by acid guanidinium thiocyanate-phenol–chloroform extraction^64,65^ was followed exactly as explained by the authors^66,67^ and the *reliable RNA preparation for Plasmodium falciparum* (denominated TRIzol extraction) was performed as described in^62^. For both, some scale adaptations were done in order to deal with samples with the smallest amount of parasites. The TRIzol extraction included an initial step of sample preservation in TRIzol at − 80 °C, for no longer than 48 h. The three commercial kits, RNeasy Micro (Qiagen), RNeasy Micro Plus (Qiagen) and Direct-zol RNA MiniPrep (Zymo Research), were used for purification of total RNA as per the manufacturer’s protocols. Additionally, for two sets of *P. falciparum* culture samples were preserved in RNAlater (RNA stabilization reagent from Qiagen) before extracted using the RNeasy Micro (RNAlater/RNeasy Micro) and the RNeasy Micro Plus kits (RNAlater/RNeasy Micro Plus), and a third set was preserved in TRI Reagent (Zymo Research) before RNA extraction with Direct-zol RNA MiniPrep kit (TRI Reagent/Direct-zol RNA MiniPrep). *P. vivax* total RNA extraction was executed using the RNeasy Micro kit (Qiagen) or Direct-zol RNA MiniPrep (Zymo Research) (Fig. 1), with the total number of Pv-iRTs from field isolates previously determined before preservation in RNAlater or TRI Reagent at − 80 °C for transportation. All *P. falciparum* and *P. vivax* RNA samples were carefully preserved precipitated in a solution of one-tenth volume of RNase-free 3 M sodium acetate pH 5.2, 2.5 volumes of absolute ethanol and one volume of isopropanol, overnight at − 20 °C and then stocked at − 80 °C. For RNA-seq, after Pv-iRT quantification, the isolate was preserved by flash freezing the sample in liquid nitrogen. For total RNA extraction, the RNeasy Micro kit (Qiagen) kit was used according to the manufacturer’s instructions. Quality control was first accessed by electrophoresing the extracted RNA samples (in triplicate) in the Agilent 2100 Bioanalyzer instrument, using the Agilent RNA 6000 Pico Kit reagents and chips, and analysed on the 2100 Expert software, according to the Agilent Technologies recommendations.

### cDNA synthesis and Quantitative PCR (qPCR)

The cDNA synthesis was carried out using 13.2 µL of RNA sample for a total reaction volume of 20 µL, using the High Capacity cDNA Reverse Transcription with RNase Inhibitor kit (Applied Biosystems), with 10 × RT buffer, 10 × Random Primers, 25x (100 mM) dNTP Mix, RNase Inhibitor and MultiScribe Reverse Transcriptase (5U.µL^-1^), according to the manufacturer´s indications. A reverse transcriptase negative control (RT-) without the enzyme was performed for all different cDNA synthesis reactions. The cDNA synthesized from *P. falciparum* and *P. vivax* RNA extracted samples was used as template for the amplification of *seryl-tRNA synthetase* (seryl-tRNA S; PFIT_0715800 and PVX_000545), *gamma-glutamylcysteine synthetase* (gamma GCS; PFIT_0919000 and PVX_099360) and *DNA repair helicase* (DNA RH; PFIT_1409400 and PVX_086025), three different *Plasmodium spp.* constitutively expressed genes. The following specific pairs of primers were used: 219 bp amplicon of Pf *seryl-tRNA S*-Fw: GAGGAATTTTACGTGTTCATCAA; Pf *seryl-tRNA S*—Rv: GATTACTTGTAGGAAAGAATCCTTC; Pv *seryl-tRNA S*—Fw: AGGGATTGCTACGTGAGCACATT; Pv *seryl-tRNA S*—Rv: GTTGCTGACTAGGTAGCCAGGCTTC; 230 bp amplicon of Pf *gamma GCS*—Fw: TGCGAATATGGATGATGAAGG; Pf *gamma GCS*—Rv: TAAGAGCAAGGAAAAGTGGT; Pv *gamma GCS*—Fw: CAGCGACCTGGACGACGAGAA; Pv *gamma GCS*—Rv: TTAGGGCTAAGAACAAAGGG; and 244 bp amplicon of Pf *DNA RH*—Fw: GCCCTTTCTATGCTACGAGA; Pf *DNA RH*—Rv: TTTTCTAGTATGGTTAATGTAGCT; Pv *DNA RH*—Fw: GCCCCTTCTACGCCACGAGG; Pv *DNA RH*—Rv: TTGTCTAGCACAGTTAGTGTAGCT. All primers were designed using Primer3 (https://primer3.ut.ee/) set on default parameters. To detect contamination in the *P. vivax* extracted RNA samples by human RNA and DNA, we also amplified the *Toll-like receptor 9* (TLR9) gene with the specific primer pair, TLR9-F: ACGTTGGATGCAAAGGGCTGGCTGTTGTAG and TLR9-R: ACGTTGGATGTCTACCACGAGCACTCATTC^23^. Power SYBR Green PCR Master Mix (Applied Biosystems) reagents were used to perform all qRT-PCR reactions and run on the Applied Biosystems 7500 Real-Time PCR System, following the company’s RT-PCR guidelines. In brief, individual reactions were set-up using 1µL of cDNA previously synthetized, 2 × Power SYBR Green PCR Master Mix, 500 nM each primer and RNase/DNase-free water to a final volume of 10 µL. The run protocol comprised the following cycling conditions: initial holding stage of 50 °C / 20 s and 95 °C/10 min; 45 × cycling stages of 95 °C/15 s, 57 °C for *P. falciparum* primers pairs or 60 °C for *P. vivax* primers pairs/30 s and 60 °C/1 min; and a 2 × melting curve stage of 95 °C/15 s and 60 °C/1 min. Calibration curves were done using five points of a standardized fivefold dilution of *P. falciparum* and *P. vivax* samples to determine the optimal primer annealing temperatures and to choose an gDNA dilution (1:25) to serve as an internal positive control in all reactions. The formation of expected PCR products was confirmed by melting curve analysis, showing a single peak. Assays were validated by running altogether the *P. falciparum* or *P. vivax* cDNA samples, their equivalent no amplification controls (RT-), a no template (absence of cDNA) control (NTC) and a positive control (*P. falciparum* or *P. vivax* gDNA). All reactions were done in quadruplicate. Efficiency was calculated using the equation E = 10^–1/slope^, where E is the theoretical efficiency and slope is determined from a standard curve plotted with y axis as Cq and the x axis as a log (Pf-iEs or Pv-iRTs) (Applied Biosystems 7500 Software v.2.0.6).

### Low input cDNA synthesis and library preparation for whole transcriptome shotgun sequencing

SMART-Seq V4 Ultra Low Input RNA kit was used for sequencing by the Clontech’s patented SMART (Switching Mechanism at 5′ End of RNA Template) technology^38–40,68^. cDNA quality, quantity and size range were evaluated through BioAnalyser platform from Agilent Technologies, Inc., using the Agilent High Sensitivity DNA Kit (cDNA, 5 to 500 pg.µL^−1^ within a size range of 50 to 7000 bp), as per manufacturer instructions. Prior to generating the final library for Illumina sequencing, the Covaris AFA system was used for controlled cDNA shearing, resulting in DNA fragments between 200 and 500 bp sizes. Instructions were followed as indicated in the SMART-Seq V4 Ultra Low Input RNA kit for sequencing user manual by Clontech Laboratories, Inc. A Takara Bio Company. cDNA output was then converted into sequencing templates suitable for cluster generation and high-throughput sequencing through the Low Input Library Prep v2 (Clontech Laboratories, Inc. A Takara Bio Company). Library quantification procedures using the Library Quantification kit (Clontech Laboratories, Inc. A Takara Bio Company) by the golden standard qPCR and Agilent's High Sensitivity DNA kit (Agilent Technologies, Inc.) were successfully completed before proceeding for the pool set-up (2 different pools of 12 samples differently indexed) at a final concentration of 2 nM for direct sequencing. The generated libraries were cluster amplified and sequenced on the Illumina platform using standard Illumina reagents and protocols for multiplexed libraries, by following their loading recommendations. The sequencing runs were performed on HiSeq 2500 sequencer on Rapid Run mode with the HiSeq Rapid Cluster Kit v2 (100 × 100) Paired End, HiSeq Rapid SBS Kit v2 (200 cycles) and HiSeq Rapid Duo cBot v2 Sample Loading kits from Illumina, Inc..

### Transcriptomic data analysis

The EuPathDB-Galaxy free, interactive, web-based platform for large-scale data analysis was used (https://eupathdb.globusgenomics.org/) ^69^. The RNA-seq raw reads were checked for quality by running Fast Quality Control (FastQC—https://www.bioinformatics.babraham.ac.uk/projectY/fastqc/). The reads were then subjected to trimming using the Trimmomatic^70^ Galaxy tool (v. 0.36.5; http://www.usadellab.org/cms/index.php?page=trimmomatic) and aligned using TopHat2^71^ (Galaxy Tool Version SAMTOOLS: 1.2; BOWTIE2: 2.1.0^72^; TOPHAT2: 2.0.14), towards the *P. vivax* P01 PlasmoDB release 38, the *P. falciparum* IT release 41 and the *Homo sapiens* UCSC hg38 (RefSeq & Gencode gene annotations embedded in HostDB release 29)^73^ reference genomes. Read count against reference genes (features) and was done by htseq-count^74^ (Galaxy Tool v. HTSEQ: default; SAMTOOLS: 1.2; PICARD: 1.134).

## Supplementary Information

Supplementary Information

## Data Availability

All data generated or analysed during this study are included in this published article (and its Supplementary Information files). Deep sequencing data was deposited in Array Express, accession number E-MTAB-8385.
