# Supplementary material for: A suitable RNA preparation methodology for whole transcriptome shotgun sequencing harvested from Plasmodium vivax-infected patients
Source: Sci Rep. 2021 Mar 3;11:5089. doi: 10.1038/s41598-021-84607-w (PMC7930272; doi:10.1038/s41598-021-84607-w)
Supplement: Supplementary file 1 — Supplementary Information [file 41598_2021_84607_MOESM1_ESM.docx]

**Supplementary Figures and Tables**

**A suitable RNA preparation methodology for Whole Transcriptome Shotgun Sequencing harvested from *Plasmodium vivax*-infected patients**

Catarina Bourgard^1^, Stefanie C. P. Lopes^2,3^, Marcus V. G. Lacerda^2,3^, Letusa Albrecht^1,4*^ and Fabio T. M. Costa^1*^

^1^ Laboratory of Tropical Diseases – Prof. Dr. Luiz Jacintho da Silva, Department of Genetics, Evolution, Microbiology and Immunology, University of Campinas – UNICAMP. Campinas, SP, Brazil.

^2^ Instituto Leônidas e Maria Deane, Fundação Oswaldo Cruz – FIOCRUZ, Manaus, AM, Brazil

^3^ Fundação de Medicina Tropical Dr. Heitor Vieira Dourado – FMT-HVD, Gerência de Malária, Manaus, AM Brazil.

^4^ Instituto Carlos Chagas, Fundação Oswaldo Cruz - FIOCRUZ, Curitiba, PR, Brazil

*** Corresponding authors:**

Prof. Dr. Fabio T. M. Costa

University of Campinas – UNICAMP

Department of Genetics, Evolution, Microbiology and Immunology

Institute of Biology

Laboratory of Tropical Diseases – Prof. Dr. Luiz Jacintho da Silva

Campinas, SP, Brazil.

E-mail: fabiotmc72@gmail.com

and

Dr. Letusa Albrecht

Laboratório de Pesquisa em Apicomplexa

Instituto Carlos Chagas, Fundação Oswaldo Cruz – FIOCRUZ

Curitiba, PR, Brazil

E-mails: [letusa.albrecht@fiocruz.br](about:blank) or [letusaa@gmail.com](about:blank)

**Supplementary Figures**

**
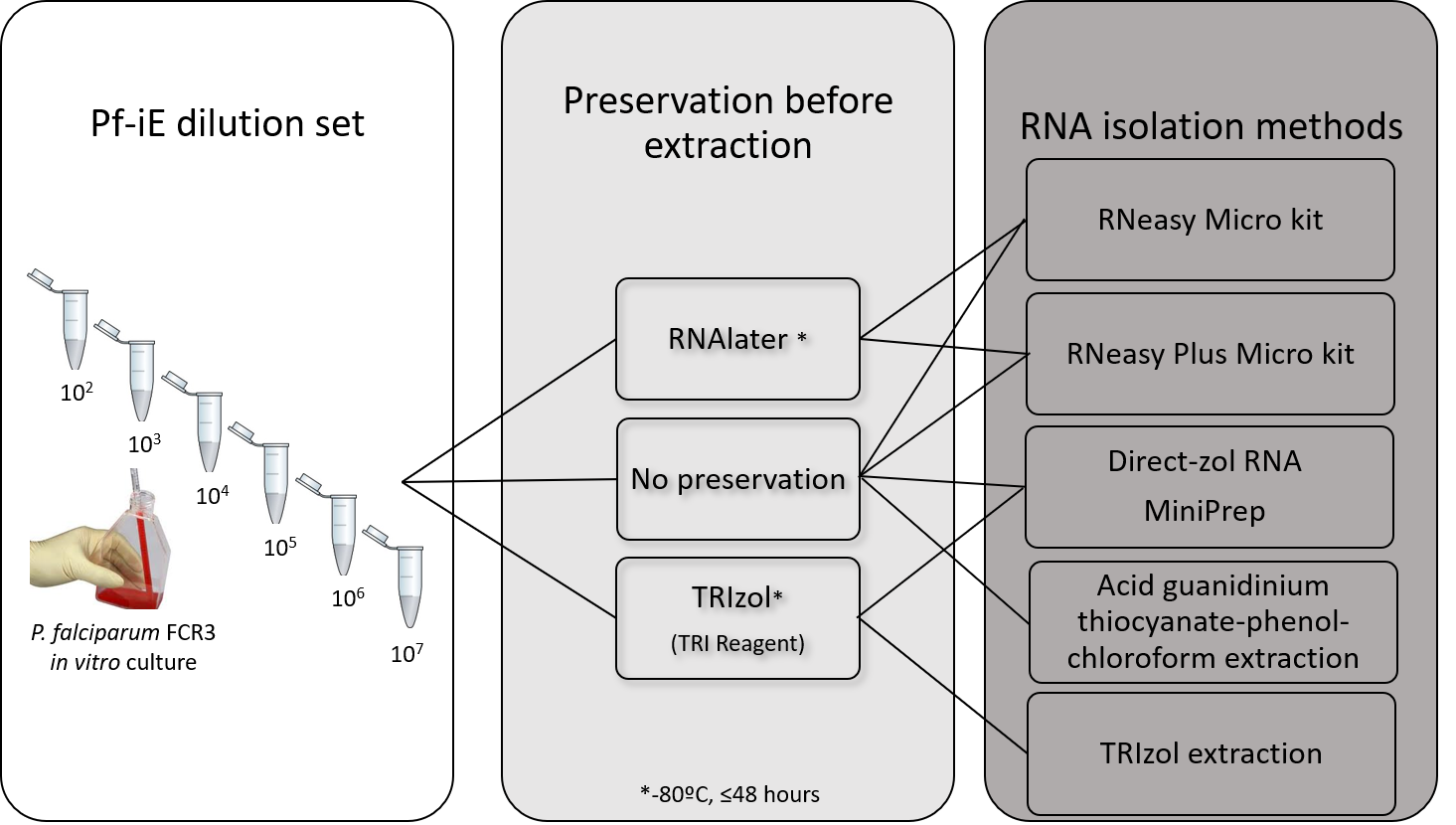
**

**Supplementary Figure S1.** ***P. falciparum* *in vitro* culture and total RNA isolation methods.** RNA was extracted from a set of *P. falciparum* infected erythrocytes (Pf-iEs) culture dilutions (10^2^ to 10^7^ in triplicate). The single-step method of RNA isolation by acid guanidinium thiocyanate-phenol-chloroform extraction [^1^](#_ENREF_1)^,^[^2^](#_ENREF_2) was directly used upon sample acquisition. An initial step of sample preservation in TRIzol at -80 ºC, for no longer than 48 h, was executed accordingly to the reliable RNA preparation for *P. falciparum* protocol [^3^](#_ENREF_3). The RNeasy Micro, RNeasy Micro Plus and Direct-zol RNA MiniPrep kits were applied as per manufacturer’s protocol. Two sets of Pf-iE dilutions were preserved in RNAlater stabilization reagent or TRI Reagent at -80 ºC, for no longer than 48 h.

**
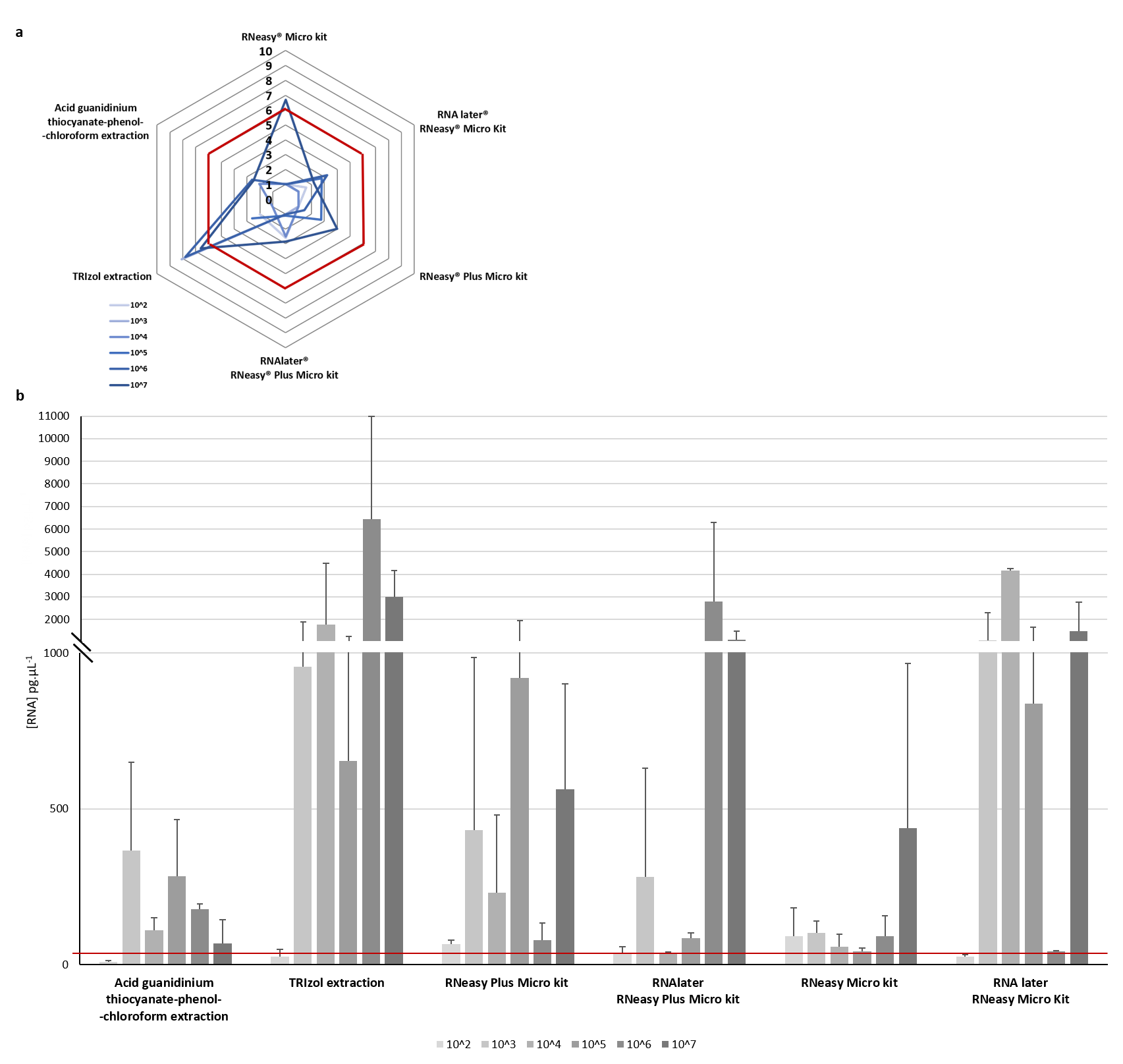
**

**Supplementary Figure S2.** ***P. falciparum* total RNA analysis.** Average RNA Integrity Number (RIN) (**a**) and quantification (limit of detection set at 50 pg.µL^-1^, dark red line) (**b**) for *P. falciparum* RNA samples extracted by different methods and analyzed on the Agilent Bioanalyzer platform. RNA with RIN ≥ 6.0 is considered suitable for RNA-seq.

**
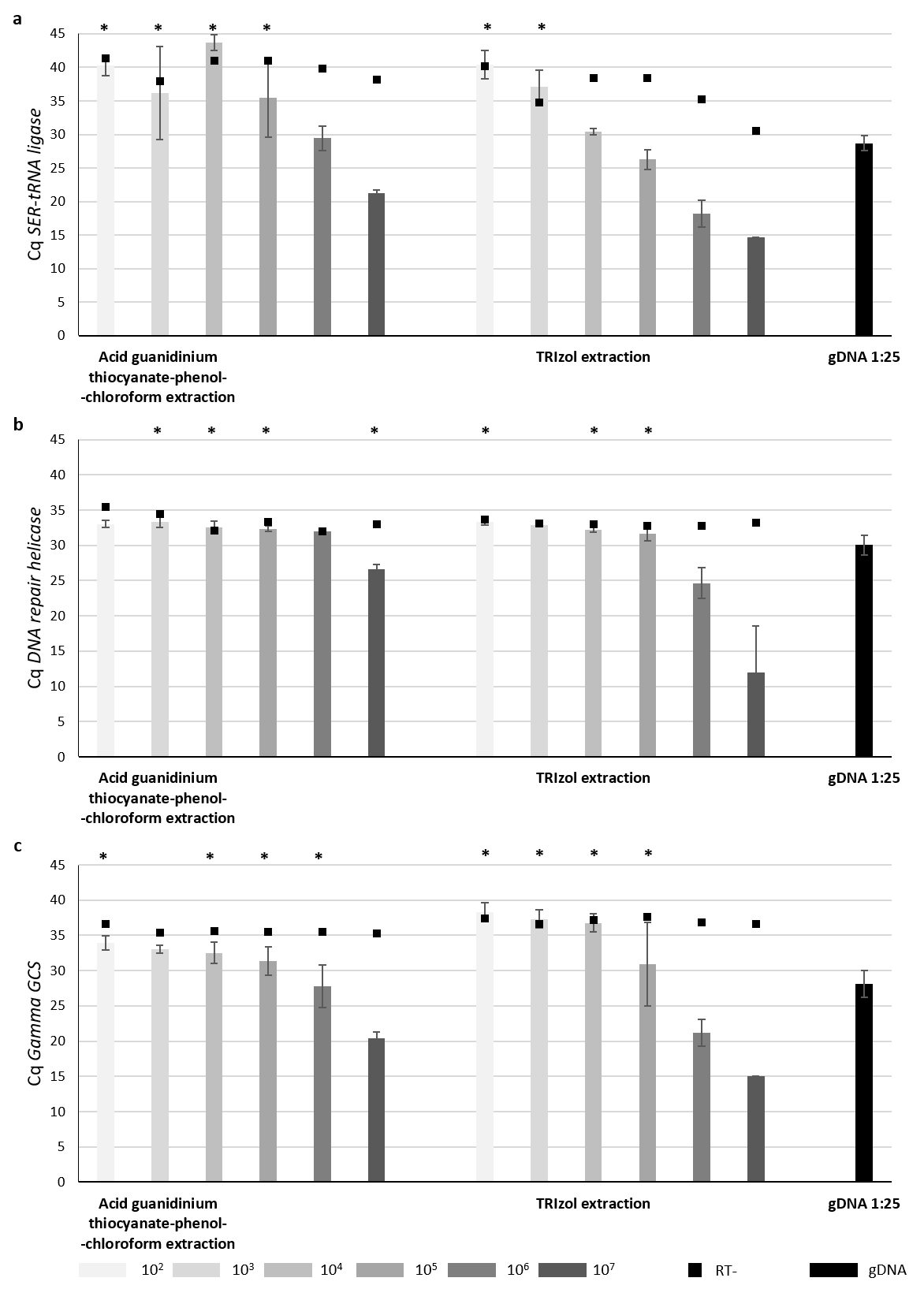
**

**Supplementary Figure S3.** **qRT-PCR of *P. falciparum* total RNA samples extracted using manual protocols.** qRT-PCR amplification of housekeeping genes *seryl-tRNA synthetase* (**a**), *DNA repair helicase* (**b**) and *gamma-glutamylcysteine synthetase* (**c**)*,* from the RNA samples of Pf-iE culture dilutions (10^2^ in light gray to 10^7^ in dark gray), extracted by each different method. Bars and error bars represent the mean Cq and standard deviations, respectively. Black square marks represent mean Cq of RT- reactions, when amplification was observed before the last (45^th^) cycle stage. Black * indicate when the mean Cq for each sample is higher than the limit of detection (3 times the Cq SDs from the correspondent RT- mean Cq).

**
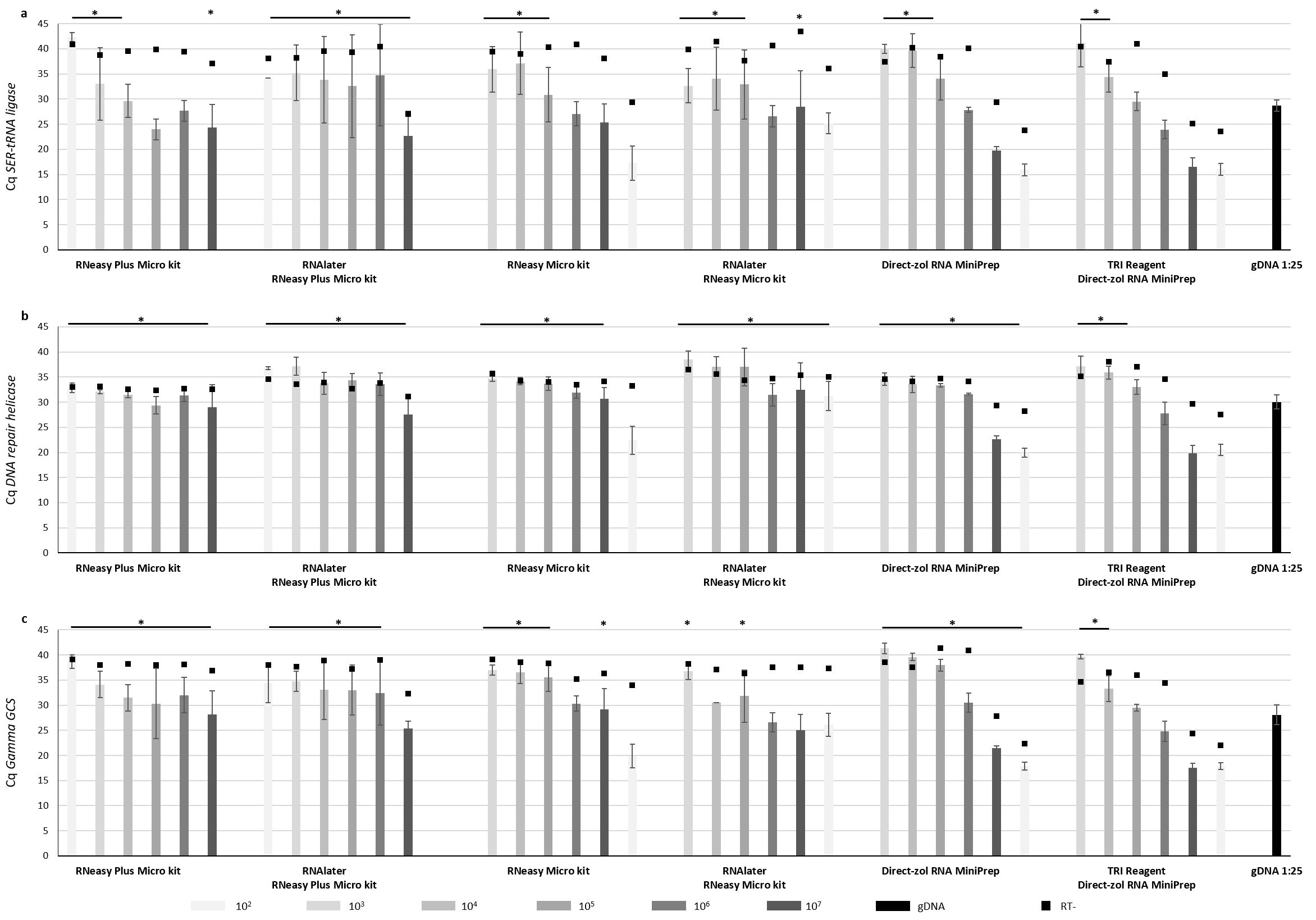
**

**Supplementary Figure S4.** **qRT-PCR of *P. falciparum* total RNA samples extracted using kits.** qRT-PCR amplification of housekeeping genes *seryl-tRNA synthetase* (**a**), *DNA repair helicase* (**b**) and *gamma-glutamylcysteine synthetase* (**c**)*,* from the RNA samples of Pf-iE culture dilutions (10^2^ in light gray to 10^7^ in dark gray), extracted by each different method. Bars and error bars represent the mean Cq and standard deviations, respectively. Black square marks represent mean Cq of RT- reactions, when amplification was observed before the last (45^th^) cycle stage. Black * indicate when the mean Cq for each sample is higher than the limit of detection (3 times the Cq SDs from the correspondent RT- mean Cq).

**
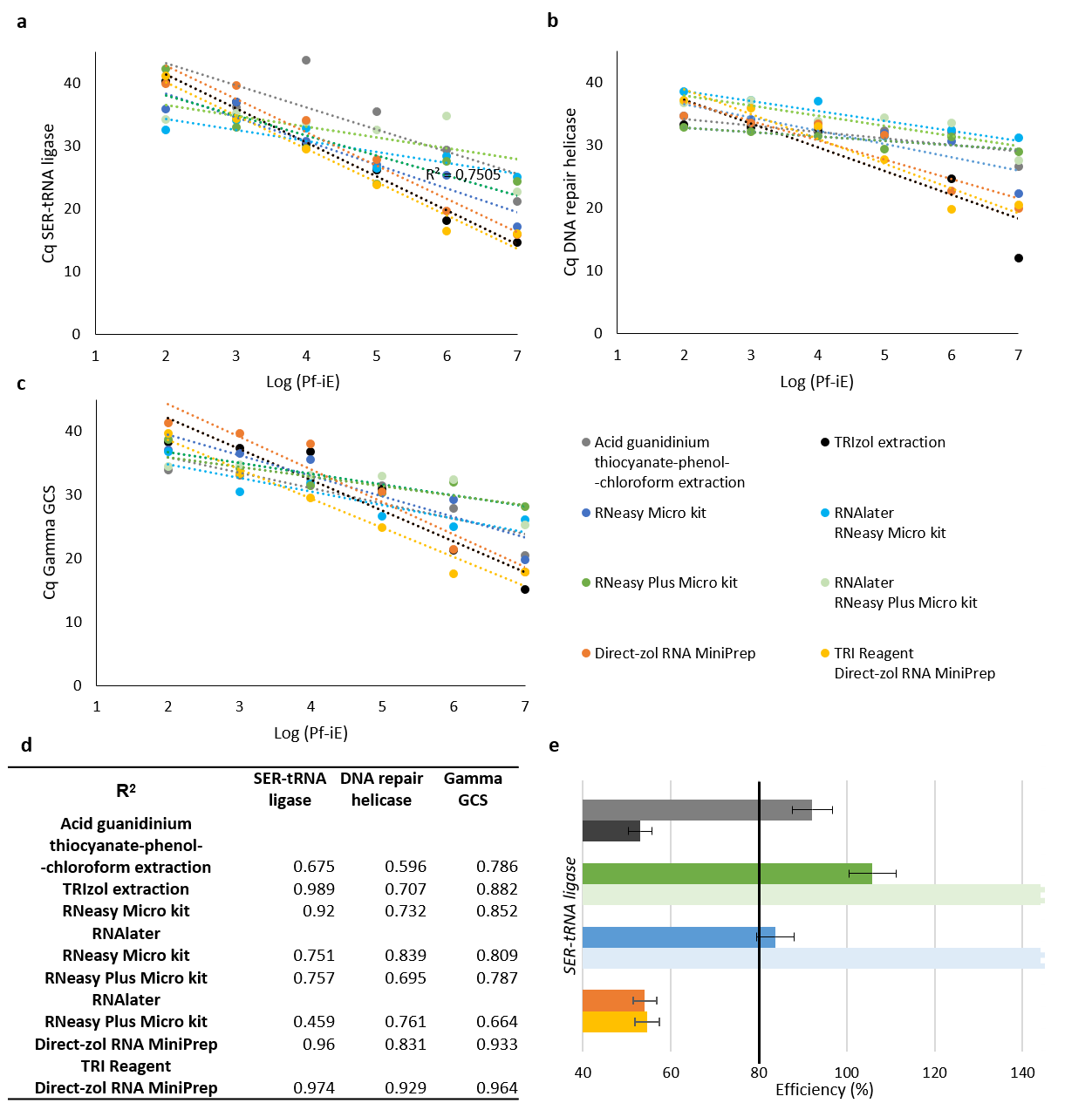
**

**Supplementary Figure S5.** **qRT-PCR coefficients of correlation and efficiency estimations for comparison of eight selected *P. falciparum* total RNA extraction methods.** Plots of parasite input (Log of the number of Pf-iE) against qRT-PCR signals (Cq) are shown for amplification of *seryl-tRNA synthetase* (**a**), *DNA repair helicase* (**b**) and *gamma-glutamylcysteine synthetase* (**c**) (Supplementary Fig. S3 and S4). Estimated correlation coefficients (R^2^) are presented on table **d**. Efficiencies were estimated from standard curves of RNA samples extracted from Pf-iE cultures on 10-fold concentration of parasites, based on six points (10^2^, 10^3^, 10^4^, 10^5^ and 10^6^ Pf-iEs). For simplicity, efficiencies for *seryl-tRNA synthetase* amplifications as presented here. *DNA repair helicase* and *gamma-glutamylcysteine synthetase* amplifications showed similar results. Error bars show a 95% confidence interval.

**
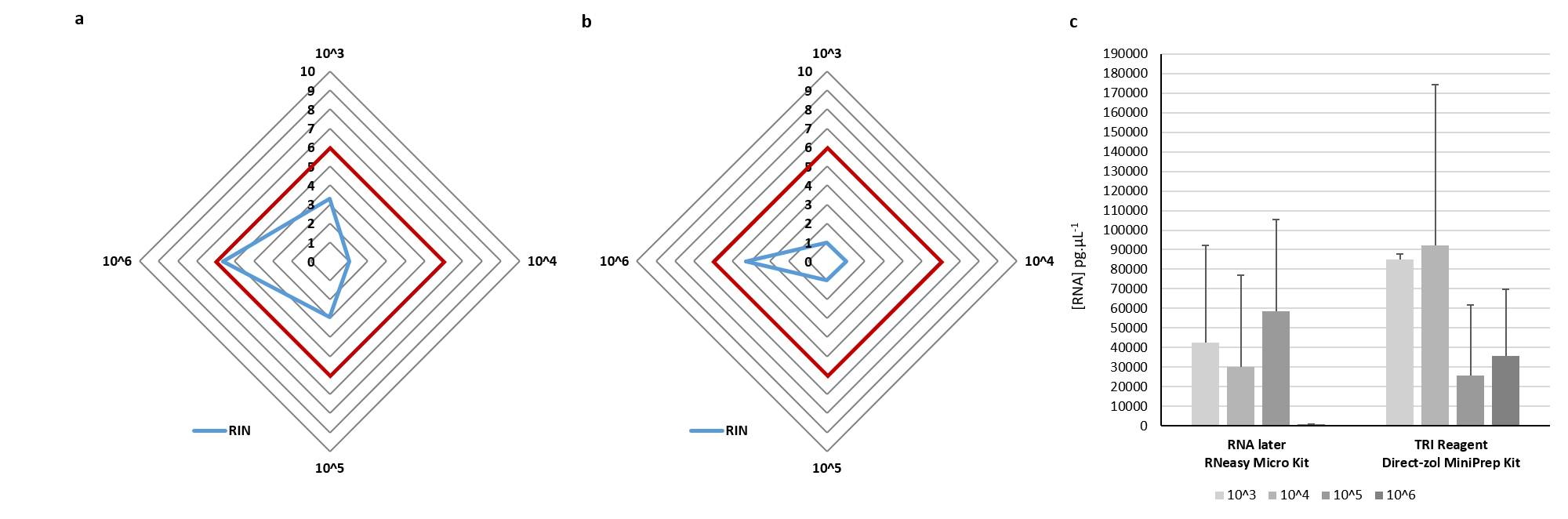
**

**Supplementary Figure S6.** ***P. vivax* total RNA analysis.** Average RIN for *P. vivax* RNA preserved in RNAlater and extracted using RNeasy Micro kit (**a**), preserved in TRI Reagent and extracted using Direct-zol MiniPrep kit (**b**), and quantification (limit of detection set at 50 pg.µL^-1^) obtained on the Agilent Bioanalyzer platform. RNA with RIN ≥ 6.0 is considered suitable for RNA-seq.


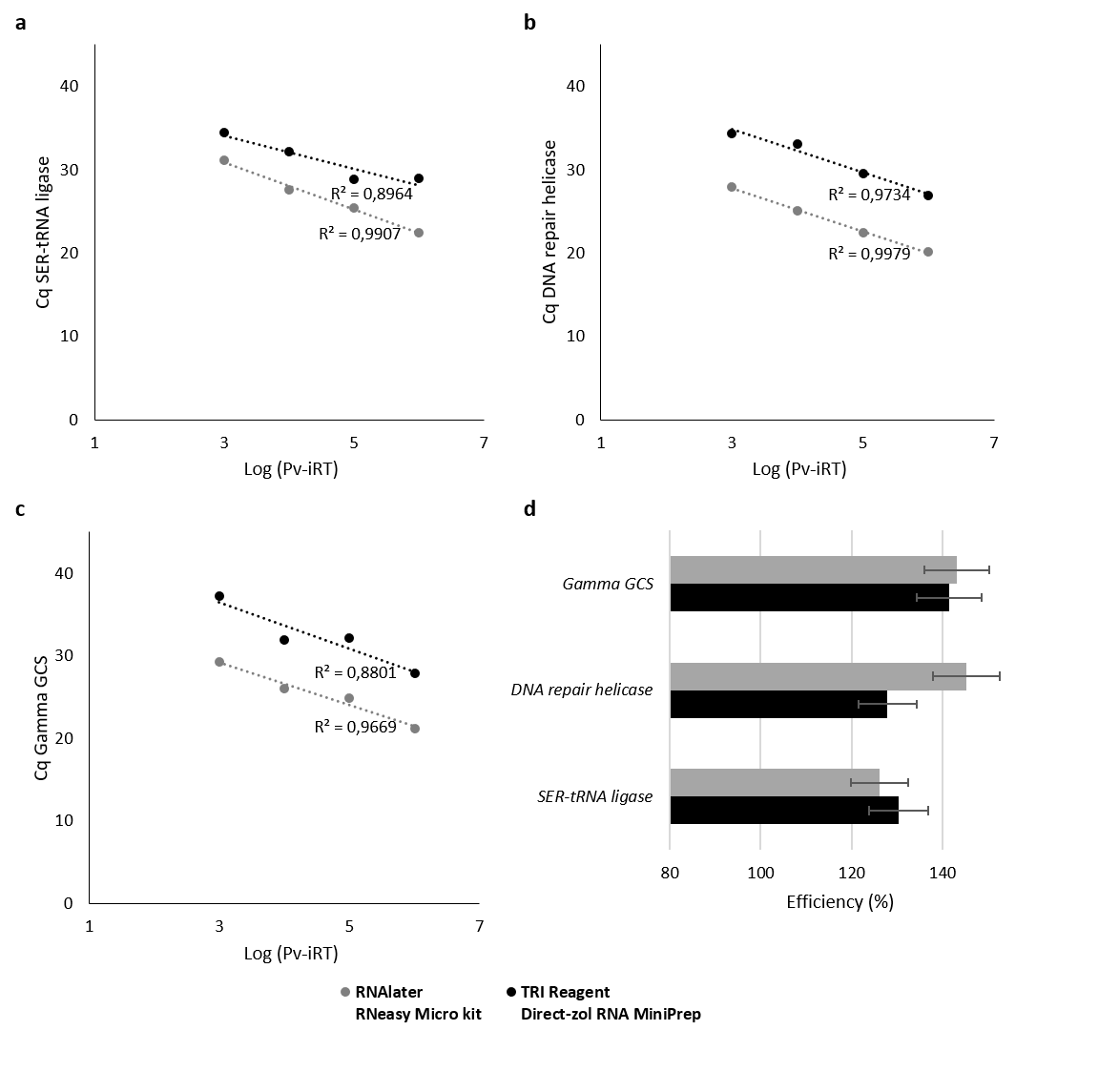
**Supplementary Figure S7. qRT-PCR efficiency estimations for comparison of two selected *P. vivax* total RNA extraction methods.** Correlation coefficients (R^2^) were estimated from plots presenting parasite input (Log of the number of Pf-iE) against qRT-PCR signals (Cq) for amplification of *seryl-tRNA synthetase* (**a**), *DNA repair helicase* (**b**) and *gamma-glutamylcysteine synthetase* (**c**) (Fig. 2). Efficiencies were estimated from standard curves of RNA samples extracted from Pv-iRT isolates on 10-fold concentration of parasites, based on four points (10^3^, 10^4^, 10^5^ and 10^6^ Pv-iRTs), using two different methods, RNAlater + RNeasy Micro kit (grey) and TRI Reagent + Direct-zol RNA MiniPrep (black). Error bars show a 95% confidence interval.

**
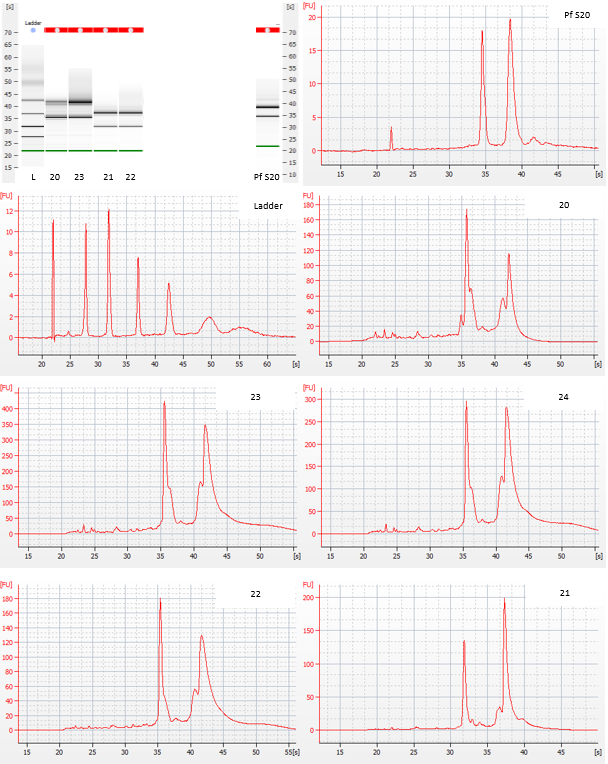
**

**Supplementary Figure S8. Bioanalyzer RNA quantity, quality and purity analysis.** Examples of the Bioanalyzer gel runs and EGRAM graphs obtained for *P. vivax* isolates 93U15 (samples 21 and 22) and 101U15 (20, 23 and 24), and *P. falciparum* S20 sample. Please, see Supplementary Table S2 for Bioanalyzer measurements after RNA extraction with RNeasy Micro kit.


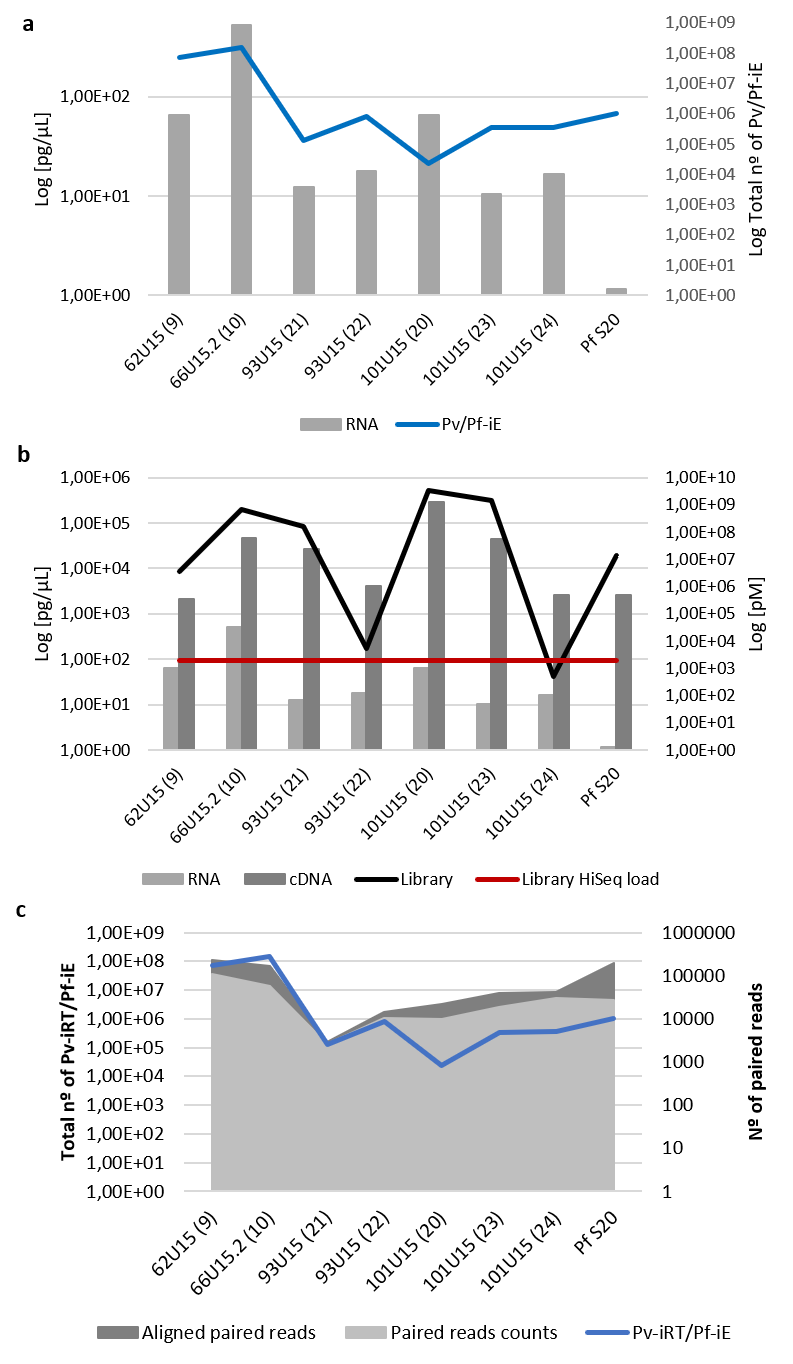


**Supplementary Figure S9.** **From *P. vivax* infected reticulocytes to alignment of paired reads sequenced.** **a.** **Comparison of number of parasite infected RBCs per sample and total RNA extraction.** Graphic representation of the number of parasitized RBCs estimated for each sample (*P. vivax* isolates and the *P. falciparum* S20 culture) manually counted (Supplementary Table S1; blue line, right log_10_ scale of the total nº of Pv-iRTs/Pf-iEs) against the respective amount of extracted tRNA (Supplementary Table S2; grey bars, log_10_ scale of concentration of RNA in pg.µL^-1^) by RNeasy Micro kit. **b.** **Quantitative representation from RNA to library generation.** The graph shows the amounts of RNA (light grey bars), cDNA (dark grey bars) (log_10_ scale in pg.µL^-1^ of the respective nucleic acids) and final libraries (black line, right log_10_ scale of pM concentration) generation per each sample for further sequencing (Supplementary Tables S2 and S3). Red line represents the optimal concentration of 2 nM recommended, for sample pool and load on the sequencer. **c. Quantitative representation between *Plasmodium* spp. infected RBCs and read alignment, mapping and count.** The area graph shows the number of parasitized RBCs estimated for each sample (*P. vivax* isolates and the *P. falciparum* S20 culture) (Supplementary Table S1; blue line, right log_10_ scale of the total nº of Pv-iRTs/Pf-iEs), the aligned and mapped trimmed reads to the correspondent *P. vivax* P01 or *P. falciparum* IT reference genomes (aligned paired reads in dark grey area) and final paired reads count to features (genes) (light grey area, right log_10_ scale of nº of paired reads) for each sample (Supplementary Table S1, S2 and S4).


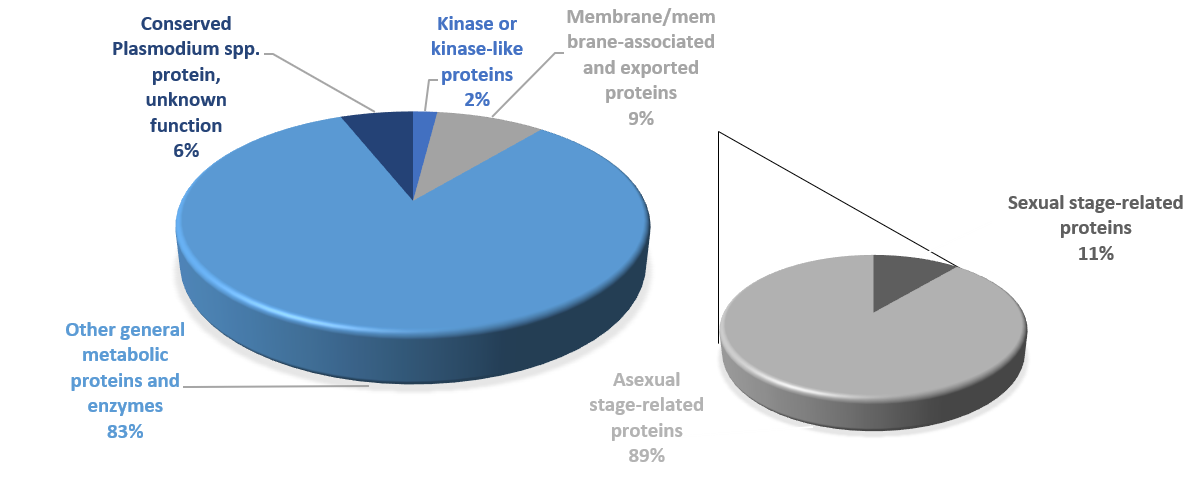


**Supplementary Figure S10. Pie charts showing the top 100 most expressed genes from RNA sequencing of *P. falciparum* field isolates**, grouped by general *Plasmodium* spp**.** metabolic proteins and enzymes (83%, light blue), membrane, membrane-associated and exported proteins (9%, light grey), conserved *Plasmodium*-like proteins of unknown function (6%, dark blue) and kinases or kinase-like proteins (2%, blue). The amplified right-hand side pie chart shows the percentage of asexual (9%, light grey) and asexual (11%, dark grey) membrane, membrane-associated and exported proteins (89%, light grey).


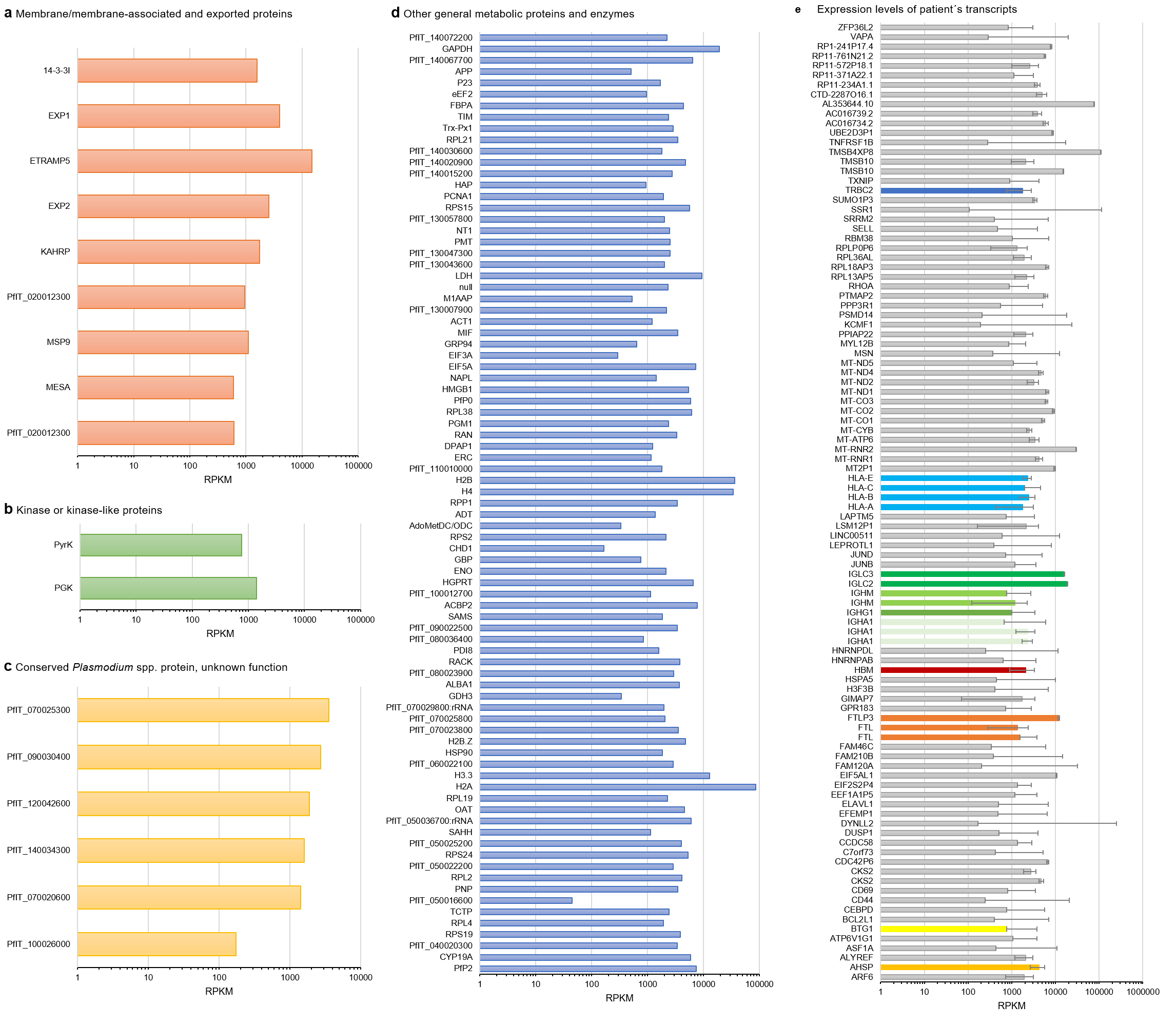


**Supplementary Figure S11. Expression levels of *P. falciparum* and human transcripts.** Expression levels in gene coverage for *P. falciparum* S20 *in vitro* culture (Supplementary Table S12) sample of **a.** membrane and membrane-associated and exported proteins, **b.** kinase or kinase-like proteins, **c.** conserved *Plasmodium* spp. proteins of unknown function, and **d.** other general metabolic proteins and enzymes. Expression levels of patient´s transcripts across all isolates are shown in **e.** (Supplementary Table S13). Error bars represent the standard deviation between RPKM values across all samples (Supplementary Tables S12 and S13).

**Supplementary Tables**

**Supplementary Table S1.** Patient profiles of *P. vivax* clinical isolates

| Patient code | Sex | Age | Parasites/µL | WBC (x10^3^/µl) | RBC  (x10^6^/µL) | HGB (g/dL) | HCT (%) | PLT  (x10^3^/µL) | LYM  (x10^3^/µL) | NEUT (x10^3^/µL) |
| --- | --- | --- | --- | --- | --- | --- | --- | --- | --- | --- |
| 62U15 | F | 32 | 9212 | 5.5 | 4.04 | 11 | 34.6 | 42 | 1.2 | 3.8 |
| 66U15 | M | 45 | 19000 | 11.2 | 5.17 | 14 | 45 | 260 | 0.6 | 9.4 |
| 93U15 | M | 44 | 5627 | 3.4 | 4.43 | 11.8 | 39.4 | 45 | F1* 0,5 | 2.3 |
| 101U15 | M | 32 | 4439 | 4.6 | 5.16 | 15 | 47.4 | 93 | 0.8 | 3.7 |

Summary of patients and clinical isolates information after collection during field work at vivax malaria endemic area. M: Male; F: Female; WBC: White Blood Cells; RBC: Red Blood Cells; HGB: hemoglobin; HCT: Hematocrit; PLT: Platelets; LYM: Lymphocytes; NEUT: Neutrophils. For information on hemogram flags please see https://www.sysmex.co.za/fileadmin/media/f112/SEED/English/Sysmex_SEED_6_2013_Haematology_Results_Interferences__Flagging_and_

Interpretation _-_Part_II_EN.pdf.

**Supplementary Table S2.** Bioanalyzer measurements after RNA extraction with RNeasy Micro kit.

| Isolate code | Pv-iRT sample code | RNA  [pg/uL] | RIN |
| --- | --- | --- | --- |
| 62U15 | 9 | 65.531 | 8.4 |
| 66U15.2 | 10 | 522 | N/A |
| 93U15 | 21 | 12.437 | N/A |
|  | 22 | 17.819 | 9.3 |
| 101U15 | 20 | 65.81 | N/A |
|  | 23 | 10.465 | N/A |
|  | 24 | 16.529 | N/A |
| Pf S20 | 18 | 1.164 | N/A |

RIN: RNA Integrity Number; N/A: undetermined.

**Supplementary Table S3.** Bioanalyzer measurements after RNA extraction with RNeasy Micro kit, and quality and quantity controls after cDNA library generation and amplification.

| **Sample Code** | **Pv-iRT sample code** | **cDNA** [pg/uL] | **cDNA** [ng/uL] | **V to 20ng** (µL) | **Total cDNA** [ng/75uL] | **V_T_ to 20ng/reaction from 75µL after shearing** (µL) | **V Elution Buffer** (µL) | **Total cDNA** [ng/reaction] | **Nº of PCR cycles for Low Input Library amplification** | **Fragment average size** (bp) | **Molarity** [pmol/L] |
| --- | --- | --- | --- | --- | --- | --- | --- | --- | --- | --- | --- |
| **62U15** | 9 | 2149.92 | 2.150 | 9.30 | 161.244 | 12.0 | - | 0.344 | 9 | 376 | 4.52E+04 |
| **66U15.2** | 10 | 46864.8 | 46.865 | 0.43 | 3514.859 | 3.2 | 6.8 | 20 | 6 | 356 | 1.01E+05 |
| **93U15** | 21 | 26400.6 | 26.401 | 0.76 | 1980.044 | 5.7 | 4.3 | 20 | 6 | 388 | 4.50E+05 |
|  | 22 | 4153.18 | 4.153 | 4.82 | 311.489 | 12.0 | - | 0.66 | 8 | 259 | 4.23E+02 |
| **101U15** | 20 | 290901 | 290.901 | 0.07 | 21817.556 | 0.5 | 9.5 | 20 | 6 | 364 | 1.71E+05 |
|  | 23 | 45498.6 | 45.499 | 0.44 | 3412.397 | 3.3 | 6.7 | 20 | 6 | 361 | 5.37E+04 |
|  | 24 | 2627.92 | 2.628 | 7.61 | 197.094 | 12.0 | - | 0.42 | 9 | 589 | 4.36E+03 |
| **Pf S20** | 18 | 2566.13 | 2.566 | 7.79 | 192.460 | 12.0 | - | 0.41 | 9 | 351 | 4.19E+04 |

*P. falciparum* S20 Brazilian field isolate (~10^6^ Pf-iE) resistant to chloroquine [^4^](#_ENREF_4) was used as control during all RNA-seq methodology. RIN: RNA Integrity Number; N/A: undetermined; V: Volume; V_T_: Total Volume; bp: base pairs.

**Supplementary Table S4.** Library quantification by qPCR and sample pool for Illumina HiSeq 2500 load and run.

| **Sample Code** | **Pv-iRT sample code** |  | **Dilution** | **Cq Mean** | **Cq Error** | **Mean** [pM] | **Error**  [pM] | **Total**  [pM] | **Total**  [nM] | ***447bp from DNA fragments** | **Library Concentration** [nM] | **Dilution** | | **V at 2nM** (µL) | **V ddH_2_O** (µL) |
| --- | --- | --- | --- | --- | --- | --- | --- | --- | --- | --- | --- | --- | --- | --- | --- |
| **62U15** | 9 |  | 1.00E-06 | 11.6 | 0.63 | 3.03E+00 | 1.48E+00 | 3.03E+06 | 3030.00 | 1.35E+09 | 3602.154 | dil -3 | 3.6021543 | 3.89 | 3.11 |
| **66U15.2** | 10 |  | 1.00E-09 | 14.25 | 0.84 | 5.29E-01 | 3.66E-01 | 5.29E+08 | 528800.00 | 2.36E+11 | 663970.8 | dil -5 | 6.6397079 | 2.11 | 4.89 |
| **93U15** | 21 |  | 1.00E-09 | 16.03 | 0.79 | 1.43E-01 | 6.84E-02 | 1.43E+08 | 1.42E05 | 6.37E+10 | 164284 | dil -4 | 16.4284 | 0.85 | 6.15 |
|  | 22 |  | 1.00E-04 | 14.77 | 0.34 | 2.99E-01 | 7.21E-02 | 2.99E+03 | 2.99 | 1.34E+06 | 5.15862 | dil 0 | 5.15862 | 2.71 | 4.29 |
| **101U15** | 20 |  | 1.00E-09 | 11.79 | 0.43 | 2.66E+00 | 8.74E-01 | 2.66E+09 | 2.66E06 | 1.19E+12 | 3266538 | dil -6 | 3.26654 | 4.29 | 2.71 |
|  | 23 |  | 1.00E-09 | 12.81 | 0.74 | 1.16E+00 | 5.44E-01 | 1.16E+09 | 1.16E06 | 5.19E+11 | 1436343 | dil -5 | 14.3634 | 0.97 | 6.03 |
|  | 24 |  | 1.00E-04 | 17.56 | 0.42 | 4.11E-02 | 1.07E-02 | 4.11E+02 | 0.41 | 1.84E+05 | 0.48873 | dil 0 | 0.48873 | 28.65 | N/A |
| **Pf S20** | 18 |  | 1.00E-07 | 13.20 | 0.90 | 1.09E+00 | 5.77E-01 | 1.09E+07 | 10900.00 | 4.87E+09 | 13881.2 | dil-3 | 13.881197 | 1.01 | 5.99 |

*P. falciparum* S20 Brazilian field isolate (~10^6^ Pf-iE) resistant to chloroquine [^4^](#_ENREF_4) was used as control during all RNA-seq methodology. RIN: RNA Integrity Number; N/A: undetermined; V: Volume; V_T_/sample = 7.0 µL; bp: base pairs.

**Supplementary Table S5.** Raw data (reads) description and statistics output from RNA-sequencing after performing quality control and trimming.

| **Sample Code** | **Sample**  **ID** | **Lane** | **Index** | **Filename** | **Total Sequences (FastQC)** | **Sequence length** | **%GC** | **Total nº of reads after trimming (%)**  **(Trimmomatic)** |  |
| --- | --- | --- | --- | --- | --- | --- | --- | --- | --- |
| **62U15** | 9 | 2 | TTAGGC | 9_S14_L001_R1_001.fastq.gz 9_S15_L001_R2_001.fastq.gz | 567222 567222 | 101 101 | 42 43 | 546571  546571  (96.4%) |  |
| **66U15.2** | 10 | 2 | GCCAAT | 10_S17_L001_R1_001.fastq.gz 10_S17_L001_R2_001.fastq.gz | 396869 396869 | 101 101 | 49 49 | 393248  393248  (99.1%) |  |
| **93U15** | 21 | 1 | CAGATC | 21_S7_L001_R1_001.fastq.gz 21_S7_L001_R2_001.fastq.gz | 481872 481872 | 101 101 | 49 49 | 475794  475794  (98.7%) |  |
|  | 22 | 1 | TAGCTT | 22_S10_L001_R1_001.fastq.gz 22_S10_L001_R2_001.fastq.gz | 225383 225383 | 101 101 | 43 45 | 214949  214949  (95.4%) |  |
| **101U15** | 20 | 1 | GCCAAT | 20_S6_L001_R1_001.fastq.gz 20_S6_L001_R2_001.fastq.gz | 231269 231269 | 101 101 | 46 47 | 226387  226387  (97.9%) |  |
|  | 23 | 1 | ACTTGA | 23_S8_L001_R1_001.fastq.gz 23_S8_L001_R2_001.fastq.gz | 761780 761780 | 101 101 | 48 49 | 754181  754181  (99.0%) |  |
|  | 24 | 1 | GATCAG | 24_S9_L001_R1_001.fastq.gz 24_S9_L001_R2_001.fastq.gz | 493265 493265 | 101 101 | 47 48 | 475848  475848  (96.5%) |  |
| **Pf S20** | 18 | 2 | TGACCA | 18_S15_L001_R1_001.fastq.gz 18_S15_L001_R2_001.fastq.gz | 383309 383309 | 101 101 | 30 30 | 379684  379684  (99.1%) |  |

Quality control and trimming of the raw data (paired-end reads) was performed using FastQC and Trimmomatic as recommended (see methods section). All samples passed FastQC quality controls with none of the sequences flagged as poor quality. The sample reference used for all field isolates was *P. vivax* P01 reference genome [^5^](#_ENREF_5) and for *P. falciparum* S20 Brazilian field isolate resistant to chloroquine [^4^](#_ENREF_4) was *P. falciparum* IT reference genome for downstream analysis.

**Supplementary Table S6.** Alignment and mapping summary report for *P. vivax* clinical isolates RNA sequenced.

|  | **Sample Code** | | | | | | | |  |
| --- | --- | --- | --- | --- | --- | --- | --- | --- | --- |
|  | **62U15** | **66U15** | **93U15** | | | **101U15** | | | ***P. falciparum* S20** |
|  |  |  | **93U15** (21) | **93U15** (22) | **101U15** (20) | | **101U15** (23) | **101U15** (24) |  |
| **Left reads** |  |  |  |  |  | |  |  |  |
| Input | 546571 | 393248 | 475794 | 214949 | 226387 | | 754181 | 475848 | 379684 |
| Mapped (% of input) | 278618 (51.0%) | 185087 (47.1%) | 5239 (1.1%) | 18875 (8.8%) | 26956 (11.9%) | | 47834 (6.3%) | 51521 (10.8%) | 216646 (57.1%) |
| Multiple alignments (% of mapped) | 7705 (2.8%) | 2873 (1.6%) | 1118 (21.3%) | 1108 (5.9%) | 1810 (6.7%) | | 2019 (4.2%) | 2624 (5.1%) | 19189 (8.9%) |
| Multiple alignments (>20) | 89 | 15 | 27 | 20 | 55 | | 38 | 57 | 114 |
| **Right reads** |  |  |  |  |  | |  |  |  |
| Input | 546571 | 393248 | 475794 | 214949 | 226387 | | 754181 | 475848 | 379684 |
| Mapped | 277677 (50.8%) | 184911 (47.0%) | 6457 (1.4%) | 19727 (9.2%) | 26585 (11.7%) | | 48540 (6.4%) | 51689 (10.9%) | 216739 (57.1%) |
| Multiple alignments | 8896 (3.2%) | 3363 (1.8%) | 2053 (31.8%) | 2171 (11.0%) | 2256 (8.5%) | | 3527 (7.3%) | 4078 (7.9%) | 19802 (9.1%) |
| Multiple alignments (>20) | 90 | 15 | 27 | 20 | 55 | | 38 | 57 | 115 |
| **Overall read mapping rate** | **50.9%** | **47,0%** | **1.2%** | **9.0%** | **11.8%** | | **6.4%** | **10.8%** | **57.1%** |
|  |  |  |  |  |  | |  |  |  |
| **Aligned pairs** | **232635** | **169102** | **2914** | **14723** | **21599** | | **39428** | **41269** | **198354** |
| Multiple alignments  (% of aligned pairs) | 5007 (2.2%) | 2021 (1.2%) | 177 (6.1%) | 279 (1.9%) | 676 (3.1%) | | 551 (1.4%) | 769 (1.9%) | 17007 (8.6%) |
| Discordant alignments  (% of aligned pairs) | 7591 (3.3%) | 1297 (0.8%) | 159 (5.5%) | 215 (1.5%) | 393 (1.8%) | | 416 (1.1%) | 580 (1.4%) | 2415 (1.2%) |
| **Concordant pair alignment rate** | **41.2%** | **42.7%** | **0.6%** | **6.7%** | **9.4%** | | **5.2%** | **8.6%** | **51.6%** |
| **Coverage** | **1.60** | **1.16** | **0.02** | **0.10** | **0.15** | | **0.27** | **0.28** | **1.71** |
| **RPKM** | **35.370** | **25.727** | **0.590** | **1.948** | **3.563** | | **5.915** | **4.415** | **80.939** |

Data table summarizing the alignment and mapping results against the *P. vivax* P01 reference genome obtained using TopHat2 on our raw data (reads) for the clinical isolates that were sequenced. Both pair of reads (left and right) were mapped, checked for multiple alignments and concordance of pair alignment. Same analysis is shown for our control sample of *P. falciparum* S20 against the *P. falciparum* IT reference genome. The estimation of sequencing coverage was calculated using the Lander/Waterman equation C = 2LN/G, where C stands for coverage, 2*L is paired-end (2) end sequencing multiplied by L, the read length (100 bp), N is the number of reads aligning to genes (features) and G is the haploid genome length, 29.05 Mbp for *P. vivax* P01 and 23.18 Mbp for *P. falciparum* IT reference genomes (<https://www.illumina.com/documents/products/technotes/technote_coverage_calculation.pdf>). RPKM (reads per kilo base per million mapped reads) was calculated using the following equation: RPKM = (C * 10^3^ *10^6^) / (N * L), where C stands for the number of reads per sample, N is the total number of reads obtained in the experiment and L is the total exon length (in bp), 12,898,530 bp for *P. vivax* P01 and 12,355,032 bp for *P. falciparum* IT reference genomes.

**Supplementary Table S7.** Alignment and mapping summary report for the human reference genome of the clinical isolates RNA sequenced.

|  | **Sample Code** | | | | | | |
| --- | --- | --- | --- | --- | --- | --- | --- |
|  | **62U15** | **66U15** | **93U15** | | | **101U15** | |
|  |  |  | **93U15** (21) | **93U15** (22) | **101U15** (20) | **101U15** (23) | **101U15** (24) |
| **Left reads** |  |  |  |  |  |  |  |
| Input | 546571 | 393248 | 475794 | 214949 | 226387 | 754181 | 475848 |
| Mapped (% of input) | 17892 (3.3%) | 65596 (16.7%) | 8714 (1.8%) | 12537 (5.8%) | 37220 (16.4%) | 23030 (3.1%) | 41728 (8.8%) |
| Multiple alignments (% of mapped) | 9324 (52.1%) | 35234 (53.7%) | 4026 (46.2%) | 8430 (67.2%) | 33160 (89.1%) | 9134 (39.7%) | 26870 (5.1%) |
| Multiple alignments (>20) | 467 | 459 | 255 | 297 | 4271 | 412 | 787 |
| **Right reads** |  |  |  |  |  |  |  |
| Input | 546571 | 393248 | 475794 | 214949 | 226387 | 754181 | 475848 |
| Mapped | 19640 (3.6%) | 67326 (17.1%) | 10449 (2.2%) | 13417 (6.2%) | 36414 (16.1%) | 25785 (3.4%) | 42124 (8.9%) |
| Multiple alignments | 112558 (57.3%) | 36563 (54.3%) | 5616 (53.7%) | 9298 (68.3%) | 32487 (89.2%) | 11680 (45.3%) | 27612 (65.5.9%) |
| Multiple alignments (>20) | 467 | 459 | 255 | 297 | 4270 | 415 | 791 |
| **Overall read mapping rate** | **3.4%** | **16,9%** | **2.0%** | **6.0%** | **16.3%** | **3.2%** | **8.8%** |
|  |  |  |  |  |  |  |  |
| **Aligned pairs** | **7380** | **58857** | **2377** | **8154** | **29685** | **12514** | **30931** |
| Multiple alignments  (% of aligned pairs) | 2756 (37.3%) | 31572 (53.6%) | 1147 (48.3%) | 5558 (68.2%) | 27055 (91.1%) | 4569 (36.5%) | 20010 (64.7%) |
| Discordant alignments  (% of aligned pairs) | 1228 (16.6%) | 1210 (2.1%) | 1726 (30.5%) | 505 (6.2%) | 1675 (5.6%) | 1353 (10.8%) | 1423 (4.6%) |
| **Concordant pair alignment rate** | **1.1%** | **14.7%** | **0.3%** | **3.6%** | **12.4%** | **1.5%** | **6.2%** |
| **Coverage** | **0.0005** | **0.0038** | **0.0002** | **0.0005** | **0.0019** | **0.0008** | **0.002** |
| **RPKM** | **0.885** | **2.393** | **0.785** | **0.680** | **0.532** | **1.844** | **0.859** |

Data table summarizing the alignment and mapping results against the human reference genome obtained using TopHat2 on our raw data (reads) for the clinical isolates that were sequenced. Both pair of reads (left and right) were mapped, checked for multiple alignments and concordance of pair alignment. The estimation of sequencing coverage was calculated using the Lander/Waterman equation C = 2LN/G, where C stands for coverage, 2*L is paired-end (2) end sequencing multiplied by L, the read length (100 bp), N is the number of reads aligning to genes (features) and G is the haploid genome length, 3099.75 Mbp for *H. Sapiens* GRCh38 reference genome (<https://www.illumina.com/documents/products/technotes/technote_coverage_calculation.pdf>). RPKM (reads per kilo base per million mapped reads) was calculated using the following equation: RPKM = (C * 10^3^ *10^6^) / (N * L), where C stands for the number of reads per sample, N is the total number of reads obtained in the experiment and L is the total exon length (in bp), 125,343,752 bp for *H. Sapiens* GRCh38 reference genomes.

**Supplementary Table S8.** Htseq-count read alignment and feature count summary report for *P. vivax* field isolates RNA sequenced.

| **Sample code**  **Reads alignment** | **62U15** | **66U15** | **93U15** | | | **101U15** | | | ***P. falciparum* S20** |
| --- | --- | --- | --- | --- | --- | --- | --- | --- | --- |
|  |  |  | **93U15** (21) | **93U15** (22) | **101U15** (20) | | **101U15** (23) | **101U15** (24) |  |
| genes (features) | 109353 | 56509 | 2629 | 10505 | 9757 | | 18645 | 30933 | 27460 |
| ambiguous | 34993 | 18179 | 361 | 1138 | 1899 | | 5259 | 4141 | 26265 |
| not unique | 77656 | 21685 | 20756 | 25715 | 20807 | | 32626 | 45672 | 85174 |

Data table summarizing the read count to genes against *P. vivax* P01 reference genome obtained using htseq-count on our aligned and mapped data (reads) for the clinical isolates that were sequenced. For all samples only good quality alignment were considered. Same analysis is shown for *P. falciparum* S20 against the *P. falciparum* IT reference genome.

**Supplementary Table S9.** Htseq-count read alignment and feature count summary report for the human reference genome of the *P. vivax* field isolates RNA sequenced.

| **Sample code**  **Reads alignment** | **62U15** | **66U15** | **93U15** | | **101U15** | | |
| --- | --- | --- | --- | --- | --- | --- | --- |
|  |  |  | **93U15** (21) | **93U15** (22) | **101U15** (20) | **101U15** (23) | **101U15** (24) |
| genes (features) | 6177 | 2906 | 2046 | 1828 | 1650 | 3418 | 3835 |
| ambiguous | 5078 | 28039 | 5295 | 2979 | 3063 | 14407 | 13575 |
| not unique | 199881 | 227493 | 90803 | 111750 | 392435 | 159717 | 269137 |

Data table summarizing the read count to genes against the human reference genome obtained using htseq-count on our aligned and mapped data (reads) for the clinical isolates that were sequenced. For all samples only good quality alignment were considered.

**Supplementary Table S10.** Agreement between the 93U15 and 101U15 technical replicates and between all the biological replicates for *P. vivax* field isolates RNA sequenced.

| Comparison | | Number of exons in common | Kappa | SE of Kappa | 95% CI |
| --- | --- | --- | --- | --- | --- |
| **93U15**  21 | **93U15**  22 | 1095 | 0.304 | 0.009 | 0.287 – 0.322 |
| **101U15**  20 | **101U15**  23 | 3221 | 0.491 | 0.007 | 0.477 – 0.505 |
| 20 | 24 | 2771 | 0.494 | 0.007 | 0.479 – 0.508 |
| 23 | 24 | 3526 | 0.526 | 0.007 | 0.512 – 0.539 |
| **62U15**  **62U15**  **62U15**  **62U15**  **62U15**  **62U15**  **66U15**  **66U15**  **66U15**  **66U15**  **66U15**  **93U15** (21)  **93U15** (21)  **93U15** (21)  **93U15** (22)  **93U15** (22)  **93U15** (22) | **66U15**  **93U15** (21)  **93U15** (22)  **101U15** (20)  **101U15** (23)  **101U15** (24)  **93U15** (21)  **93U15** (22)  **101U15** (20)  **101U15** (23)  **101U15** (24)  **101U15** (20)  **101U15** (23)  **101U15** (24)  **101U15** (20)  **101U15** (23)  **101U15** (24) | 7172  1760  3293  3746  5081  4267  1697  3194  3610  4895  3884  1239  1487  1325  2158  2665  15324 | 0.636  0.172  0.330  0.365  0.476  0.401  -0.768  0.348  0.379  0.488  0.399  0.309  0.268  0.037  0.446  0.434  0.297 | 0.006  0.004  0.006  0.006  0.006  0.006  0.005  0.006  0.006  0.006  0.006  0.008  0.007  0.004  0.008  0.007  0.009 | 0.625 – 0.647  0.164 – 0.181  0.320 – 0.341  0.354 – 0.377  0.464 – 0.488  0.390 – 0.413  -0.778 – -0.758  0.336 – 0.359  0.367 – 0.390  0.476 – 0.501  0.386 – 0.411  0.293 – 0.326  0.255 – 0.282  0.029 – 0.044  0.430 – 0.462  0.419 – 0.448  0.280 – 0.314 |

Data table summarizing the estimations for agreement between technical replicates 21 and 22 from isolate 93U15 and 20, 23 and 24 from isolate 101U15. Kappa coefficient was used to measure agreement [^6^](#_ENREF_6)^,^[^7^](#_ENREF_7), where values < 0 report no agreement; 0.00 - 0.20, slight agreement, 0.21 – 0.40, fair agreement, 0.41 – 0.60, moderate agreement; 0.61 – 0.80, substantial agreement and 0.81 – 1.00, almost perfect agreement. The total number of exons (n) is 17754 (PlasmoDB).

**Supplementary Table S11.** List of top 100 most expressed *P. vivax* transcripts from the RNA sequenced isolates.

| **Exon ID** | **Read counts** | **Transcript Length** (bp) |  | **RPKM** | | | | | | **Gene ID or Symbol** | **Product Description** |
| --- | --- | --- | --- | --- | --- | --- | --- | --- | --- | --- | --- |
|  |  |  | **62U15 (9)** | **66U15.2 (10)** | **93U15 (21)** | **93U15 (22)** | **101U15 (20)** | **101U15 (23)** | **101U15 (24)** |  |  |
| **Membrane/membrane-associated and exported proteins** | | | | | | | | | | | |
| exon_PVP01_1208000-E1 | 270 | 1302 | 206.1 | 113.3 | 1372.5 | 582.1 | 1500.0 | 2848.0 | 2128.0 | P47 | 6-cysteine protein |
| exon_PVP01_0415800-E1 | 258 | 8178 | 13.9 | 9.0 | 349.6 | 79.4 | 687.5 | 313.9 | 286.2 | P230 | 6-cysteine protein P230, putative |
| exon_PVP01_0216700-E2 | 409 | 1488 | 845.5 | 705.1 | 480.4 | 1018.7 | 477.3 | 766.8 | 321.0 | PVP01_0216700-E2 | Plasmodium exported protein, unknown function |
| exon_PVP01_0623100-E2 | 1834 | 3663 | 2085.1 | 1125.6 | 97.6 | 857.2 | 32.3 | 136.3 | 52.2 | PVP01_0623100-E2 | Plasmodium exported protein, unknown function |
| exon_PVP01_1147600-E2 | 400 | 1512 | 662.5 | 970.4 | 472.7 | 787.7 | 548.0 | 518.8 | 126.4 | PVP01_1147600-E2 | Plasmodium exported protein, unknown function |
| exon_PVP01_1201600-E2 | 655 | 897 | 2685.4 | 1654.1 | 1593.7 | 482.8 | 857.7 | 1510.4 | 585.8 | PVP01_1201600-E2 | Plasmodium exported protein, unknown function |
| exon_PVP01_1402800-E2 | 1017 | 714 | 5619.9 | 2399.5 | 3503.9 | 2729.5 | 1657.8 | 2247.1 | 3010.7 | PVP01_1402800-E2 | Plasmodium exported protein, unknown function |
| exon_PVP01_1251100-E2 | 366 | 4824 | 249.7 | 25.5 | 444.5 | 269.3 | 552.1 | 413.9 | 297.1 | CCp2 | LCCL domain-containing protein |
| exon_PVP01_1341700-E1 | 269 | 3921 | 176.4 | 48.1 | 91.1 | 386.6 | 664.1 | 436.5 | 280.2 | CCp3 | LCCL domain-containing protein, putative |
| exon_PVP01_0315200-E1 | 193 | 1302 | 224.4 | 107.0 | 549.0 | 498.9 | 727.3 | 657.2 | 2898.4 | CDC50A | LEM3/CDC50 family protein, putative |
| exon_PVP01_1321400-E1 | 312 | 846 | 796.4 | 542.6 | 1267.4 | 128.0 | 2238.6 | 3750.9 | 1016.4 | PVP01_1321400-E1 | leucine-rich repeat protein |
| exon_PVP01_0922200-E2 | 315 | 447 | 1840.7 | 1613.8 | 2398.6 | 1211.1 | 926.8 | 4227.5 | 2244.2 | EXP1 | exported protein 1, putative |
| exon_PVP01_0610000-E1 | 329 | 3396 | 170.3 | 354.8 | 105.2 | 350.7 | 435.7 | 378.0 | 168.8 | EXP3 | exported protein 3, putative |
| exon_PVP01_0929800-E1 | 649 | 1290 | 1428.2 | 1785.6 | 277.1 | 587.5 | 596.4 | 884.5 | 222.2 | PV1 | parasitophorous vacuolar protein 1, putative |
| exon_PVP01_0422600-E1 | 2246 | 333 | 28074.8 | 12505.0 | 5366.3 | 15606.8 | 3199.0 | 8030.3 | 3442.8 | ETRAMP11.2 | early transcribed membrane protein |
| exon_PVP01_0532300-E1 | 2869 | 438 | 28137.2 | 7560.9 | 8159.8 | 11371.0 | 8242.3 | 13268.7 | 12869.3 | ETRAMP | early transcribed membrane protein |
| exon_PVP01_0532300-E2 | 418 | 438 | 3811.5 | 898.3 | 816.0 | 5685.5 | 2026.8 | 1628.1 | 3380.9 | ETRAMP | early transcribed membrane protein |
| exon_PVP01_0616100-E1 | 1003 | 660 | 5862.9 | 136.6 | 7039.7 | 5413.6 | 6994.2 | 7455.0 | 5862.6 | P25 | ookinete surface protein P25 |
| exon_PVP01_0616000-E1 | 354 | 711 | 2205.5 | 57.6 | 1508.0 | 2284.2 | 2247.4 | 1705.0 | 470.3 | P28 | ookinete surface protein P28, putative |
| **Kinase or kinase-like proteins** | | | | | | | | | | | |
| exon_PVP01_0518400-E1 | 207 | 780 | 573.3 | 210.2 | 1374.6 | 277.6 | 607.0 | 2879.8 | 2204.7 | AK2 | adenylate kinase 2, putative |
| exon_PVP01_0721000-E1 | 493 | 1251 | 910.3 | 1625.0 | 571.4 | 865.5 | 378.5 | 712.5 | 343.7 | PGK | phosphoglycerate kinase, putative |
| exon_PVP01_1300900-E1 | 275 | 891 | 890.0 | 294.4 | 0.0 | 486.1 | 2324.8 | 2441.0 | 536.1 | NEK3 | NIMA related kinase 3, putative |
| **Conserved *Plasmodium* spp. protein, unknown function** | | | | | | | | | | | |
| exon_PVP01_0309800-E1 | 167 | 618 | 646.4 | 159.2 | 4048.2 | 1752.0 | 957.6 | 1326.9 | 2937.3 | PVP01_0309800-E1 | conserved Plasmodium protein, unknown function |
| exon_PVP01_0310300-E1 | 217 | 2895 | 170.9 | 65.1 | 987.6 | 635.8 | 470.2 | 468.0 | 412.5 | PVP01_0310300-E1 | conserved Plasmodium protein, unknown function |
| exon_PVP01_0317200-E1 | 171 | 2226 | 40.2 | 187.8 | 321.1 | 97.3 | 345.6 | 416.4 | 1330.5 | PVP01_0317200-E1 | conserved Plasmodium protein, unknown function |
| exon_PVP01_0526400-E1 | 203 | 960 | 236.0 | 34.2 | 2233.7 | 451.1 | 1171.3 | 3008.3 | 2537.7 | PVP01_0526400-E1 | conserved Plasmodium protein, unknown function |
| exon_PVP01_0822100-E1 | 212 | 651 | 943.3 | 63.0 | 2196.0 | 1164.2 | 1636.4 | 2574.1 | 2054.6 | PVP01_0822100-E1 | conserved Plasmodium protein, unknown function |
| exon_PVP01_1140300-E2 | 210 | 1368 | 126.4 | 107.9 | 261.3 | 316.6 | 865.2 | 2449.9 | 1536.4 | PVP01_1140300-E2 | conserved Plasmodium protein, unknown function |
| exon_PVP01_1345600-E1 | 150 | 1077 | 49.8 | 53.3 | 0.0 | 201.1 | 1428.7 | 2549.1 | 1286.3 | PVP01_1345600-E1 | conserved Plasmodium protein, unknown function |
| exon_PVP01_1465500-E1 | 206 | 888 | 839.3 | 120.0 | 1609.9 | 1097.4 | 533.2 | 1365.1 | 699.3 | PVP01_1465500-E1 | conserved Plasmodium protein, unknown function |
| exon_PVP01_1467500-E1 | 403 | 1950 | 626.8 | 92.5 | 1466.2 | 1055.0 | 849.8 | 1170.2 | 1396.3 | PVP01_1467500-E1 | conserved Plasmodium protein, unknown function |
| exon_PVP01_1020900-E1 | 149 | 816 | 306.9 | 110.5 | 2627.9 | 1061.5 | 217.6 | 2097.3 | 1814.8 | PVP01_1020900-E1 | conserved protein, unknown function |
| exon_PVP01_1025600-E4 | 193 | 1107 | 123.9 | 22.2 | 645.7 | 97.8 | 1817.7 | 2093.5 | 2804.9 | PVP01_1025600-E4 | conserved protein, unknown function |
| **Hypothetical proteins/unspecified product** | | | | | | | | | | | |
| exon_PVP01_MIT01200-E1 | 810 | 196 | 2372.8 | 292.8 | 9117.3 | 225382.5 | 2415.6 | 545.7 | 73359.8 | PVP01_MIT01200-E1 | unspecified product |
| exon_PVP01_1469500-E3 | 377 | 8607 | 99.8 | 130.5 | 83.0 | 188.7 | 247.5 | 78.7 | 133.2 | PVP01_1469500-E3 | hypothetical protein |
| **Other general metabolic proteins and enzymes** | | | | | | | | | | | |
| exon_PVP01_0202900-E1 | 982 | 2129 | 1097.8 | 881.7 | 335.7 | 1525.7 | 7060.7 | 251.2 | 1346.2 | PVP01_0202900-E1 | 18S ribosomal RNA |
| exon_PVP01_0504500-E1 | 9124 | 4776 | 4882.5 | 1591.0 | 3891.3 | 13443.3 | 33705.1 | 1985.8 | 6551.3 | PVP01_0504500-E1 | 28S ribosomal RNA |
| exon_PVP01_0801900-E1 | 234 | 4888 | 133.0 | 65.4 | 73.1 | 332.3 | 411.7 | 43.8 | 293.2 | PVP01_0801900-E1 | 28S ribosomal RNA |
| exon_PVP01_1113100-E1 | 1039 | 456 | 9989.5 | 3954.8 | 783.8 | 4273.9 | 778.7 | 1251.0 | 1466.6 | RPS15 | 40S ribosomal protein S15, putative |
| exon_PVP01_1459800-E2 | 646 | 414 | 6624.8 | 2673.0 | 1726.6 | 4446.0 | 143.0 | 861.2 | 2423.1 | PVP01_1459800-E2 | 40S ribosomal protein S17, putative |
| exon_PVP01_1245500-E1 | 351 | 204 | 6663.8 | 2571.7 | 7007.8 | 2653.7 | 2030.8 | 3670.3 | 5151.6 | PVP01_1245500-E1 | 40S ribosomal protein S28e, putative |
| exon_PVP01_0817300-E2 | 516 | 789 | 2471.1 | 1631.1 | 906.0 | 411.7 | 375.0 | 723.0 | 363.3 | RPS3A | 40S ribosomal protein S3A, putative |
| exon_PVP01_0906200-E2 | 510 | 786 | 2617.0 | 1334.9 | 1364.1 | 1377.5 | 527.1 | 544.3 | 303.9 | RPS4 | 40S ribosomal protein S4, putative |
| exon_PVP01_1212800-E1 | 561 | 1053 | 2185.6 | 941.9 | 339.4 | 2159.3 | 0.0 | 474.0 | 816.6 | PVP01_1212800-E1 | 40S ribosomal protein S6, putative |
| exon_PVP01_1334600-E1 | 560 | 660 | 2556.6 | 3080.2 | 0.0 | 2132.6 | 89.7 | 756.3 | 72.4 | PVP01_1334600-E1 | 60S ribosomal protein L10, putative |
| exon_PVP01_0612900-E1 | 505 | 1161 | 1525.2 | 1150.9 | 923.5 | 2331.4 | 203.9 | 337.8 | 82.3 | RPL3 | 60S ribosomal protein L3, putative |
| exon_PVP01_1463200-E1 | 350 | 1131 | 796.0 | 768.3 | 2528.0 | 765.8 | 784.9 | 1639.3 | 422.4 | ACT1 | actin, putative |
| exon_PVP01_1266500-E1 | 149 | 1527 | 148.4 | 53.7 | 234.1 | 709.1 | 503.8 | 326.9 | 1970.8 | PVP01_1266500-E1 | allantoicase, putative |
| exon_PVP01_0530800-E3 | 1074 | 1353 | 846.1 | 763.4 | 5283.0 | 720.2 | 6080.1 | 10303.7 | 6955.3 | PVP01_0530800-E3 | alpha tubulin 2, putative |
| exon_PVP01_0204400-E1 | 366 | 1344 | 465.8 | 725.8 | 265.9 | 161.1 | 1893.5 | 2308.0 | 319.9 | PVP01_0204400-E1 | chromatin assembly factor 1 protein WD40 domain, putative |
| exon_PVP01_0609100-E1 | 211 | 9798 | 48.1 | 41.0 | 73.0 | 243.1 | 102.7 | 29.1 | 165.8 | CHD1 | chromodomain-helicase-DNA-binding protein 1 homolog, putative |
| exon_PVP01_1426100-E1 | 1610 | 726 | 10002.9 | 3692.1 | 984.6 | 3579.2 | 978.2 | 1227.8 | 131.6 | ALBA1 | DNA/RNA-binding protein Alba 1, putative |
| exon_PVP01_0608300-E1 | 185 | 15804 | 4.9 | 6.2 | 67.8 | 20.6 | 179.7 | 97.0 | 190.4 | PVP01_0608300-E1 | dynein heavy chain, putative |
| exon_PVP01_0703700-E1 | 129 | 17601 | 5.8 | 2.3 | 40.6 | 0.0 | 161.4 | 48.6 | 89.6 | PVP01_0703700-E1 | dynein heavy chain, putative |
| exon_PVP01_1255900-E1 | 538 | 2499 | 493.9 | 987.3 | 0.0 | 303.3 | 94.7 | 214.0 | 76.5 | eEF2 | elongation factor 2, putative |
| exon_PVP01_0716300-E2 | 555 | 1959 | 623.9 | 1230.2 | 182.4 | 552.7 | 362.5 | 473.2 | 170.7 | BIP | endoplasmic reticulum chaperone BiP, putative |
| exon_PVP01_0909400-E1 | 534 | 1035 | 1831.9 | 1235.5 | 690.6 | 627.7 | 400.3 | 1067.9 | 646.2 | ERC | endoplasmic reticulum-resident calcium binding protein, putative |
| exon_PVP01_0816000-E2 | 985 | 1341 | 2823.3 | 1839.9 | 533.0 | 645.9 | 353.1 | 638.1 | 249.4 | ENO | enolase, putative |
| exon_PVP01_1262200-E2 | 410 | 1110 | 741.3 | 937.9 | 965.9 | 682.8 | 1279.6 | 2987.3 | 774.6 | PVP01_1262200-E2 | fructose 1,6-bisphosphate aldolase, putative |
| exon_PVP01_0716400-E1 | 531 | 1188 | 1495.6 | 296.7 | 1203.4 | 1275.9 | 1096.0 | 3121.3 | 1849.6 | GAP50 | glideosome-associated protein 50, putative |
| exon_PVP01_1244000-E2 | 2397 | 1014 | 9807.8 | 3807.6 | 4229.6 | 5338.9 | 1984.4 | 4149.1 | 2072.8 | GAPDH | glyceraldehyde-3-phosphate dehydrogenase, putative |
| exon_PVP01_1444500-E1 | 297 | 882 | 851.8 | 975.9 | 1620.9 | 736.5 | 603.9 | 1374.4 | 704.1 | HAD2 | haloacid dehalogenase-like hydrolase, putative |
| exon_PVP01_0515400-E1 | 1883 | 2073 | 2738.1 | 3163.4 | 689.6 | 1149.1 | 713.7 | 1118.0 | 345.7 | HSP70 | heat shock protein 70, putative |
| exon_PVP01_0108700-E1 | 1567 | 2247 | 2603.0 | 1550.4 | 1113.4 | 1204.6 | 342.4 | 967.9 | 1169.3 | HSP90 | heat shock protein 90, putative |
| exon_PVP01_0517400-E1 | 314 | 300 | 3676.8 | 546.5 | 2382.7 | 2887.3 | 3156.4 | 4753.9 | 6846.9 | HMGB2 | high mobility group protein B2, putative |
| exon_PVP01_0905900-E1 | 2796 | 357 | 24567.4 | 9735.6 | 14015.6 | 8491.9 | 24203.3 | 23569.9 | 63826.0 | H2B | histone 2B, putative |
| exon_PVP01_1131700-E1 | 1146 | 402 | 8839.7 | 4628.8 | 16002.9 | 1885.3 | 9863.7 | 10554.4 | 13308.8 | H2A | histone H2A, putative |
| exon_PVP01_1131600-E1 | 1358 | 411 | 13070.7 | 4567.3 | 7826.2 | 6322.5 | 5183.8 | 4250.8 | 12784.9 | H3.3 | histone H3 variant, putative |
| exon_PVP01_1138700-E1 | 361 | 411 | 2379.1 | 1176.7 | 4347.9 | 1844.0 | 3167.9 | 5031.5 | 5346.4 | H3 | histone H3, putative |
| exon_PVP01_0905800-E1 | 9373 | 312 | 131610.5 | 19573.4 | 44674.8 | 23944.8 | 54819.3 | 53938.8 | 133508.7 | H4 | histone H4, putative |
| exon_PVP01_1006300-E2 | 279 | 1404 | 560.6 | 467.1 | 1018.2 | 616.9 | 210.8 | 431.7 | 1122.8 | HIP | Hsc70-interacting protein, putative |
| exon_PVP01_1253300-E2 | 405 | 801 | 1578.0 | 1361.1 | 1338.6 | 1757.2 | 295.5 | 1246.3 | 715.6 | P23 | HSP90 co-chaperone p23, putative |
| exon_PVP01_1229400-E2 | 160 | 1005 | 124.6 | 138.7 | 355.6 | 107.7 | 1943.3 | 2518.9 | 760.5 | PVP01_1229400-E2 | lactate dehydrogenase, putative |
| exon_PVP01_1229400-E3 | 326 | 1005 | 231.4 | 195.8 | 1422.5 | 430.9 | 3062.2 | 4718.4 | 3327.2 | PVP01_1229400-E3 | lactate dehydrogenase, putative |
| exon_PVP01_1229700-E1 | 982 | 951 | 2482.7 | 3999.5 | 1879.1 | 1593.9 | 1306.9 | 1987.1 | 1456.7 | LDH | L-lactate dehydrogenase |
| exon_PVP01_1412800-E1 | 1023 | 3294 | 825.4 | 1274.1 | 108.5 | 493.0 | 179.7 | 270.6 | 58.0 | M1AAP | M1-family alanyl aminopeptidase, putative |
| exon_PVP01_1435300-E1 | 773 | 867 | 2365.7 | 2070.6 | 1236.7 | 1623.5 | 2320.9 | 4811.5 | 2369.2 | MDV1 | male development gene 1, putative |
| exon_PVP01_1412100-E1 | 941 | 1488 | 296.5 | 11.0 | 3122.4 | 1018.7 | 5090.9 | 6493.5 | 14093.2 | PVP01_1412100-E1 | meiosis-specific nuclear structural protein 1, putative |
| exon_PVP01_1467200-E1 | 460 | 7743 | 48.5 | 21.2 | 323.1 | 293.6 | 1146.5 | 515.7 | 536.7 | G377 | osmiophilic body protein G377, putative |
| exon_PVP01_0818200-E1 | 358 | 513 | 2231.5 | 2013.3 | 2786.7 | 3376.9 | 115.4 | 903.5 | 558.7 | CYP19A | peptidyl-prolyl cis-trans isomerase, putative |
| exon_PVP01_0702600-E1 | 294 | 915 | 1238.1 | 62.7 | 1171.8 | 946.6 | 905.5 | 2065.2 | 991.9 | PH | PH domain-containing protein, putative |
| exon_PVP01_1318600-E1 | 186 | 807 | 266.0 | 81.3 | 885.7 | 0.0 | 1686.7 | 2606.7 | 3433.2 | PIP3 | PhIL1 interacting protein PIP3, putative |
| exon_PVP01_1211800-E4 | 875 | 795 | 5204.8 | 1422.9 | 899.1 | 1770.5 | 223.3 | 627.9 | 661.0 | PMT | phosphoethanolamine N-methyltransferase |
| exon_PVP01_0811900-E1 | 248 | 2958 | 169.3 | 279.9 | 120.8 | 585.6 | 180.1 | 301.3 | 193.8 | PREBP | PRE-binding protein, putative |
| exon_PVP01_1110100-E1 | 324 | 825 | 614.3 | 854.5 | 2599.3 | 131.2 | 1936.9 | 4062.4 | 1447.6 | PCNA1 | proliferating cell nuclear antigen 1, putative |
| exon_PVP01_1444800-E2 | 186 | 795 | 412.5 | 525.9 | 1798.2 | 272.4 | 670.0 | 1838.8 | 1442.1 | PCNA2 | proliferating cell nuclear antigen 2, putative |
| exon_PVP01_1445700-E1 | 220 | 7449 | 37.6 | 57.2 | 191.9 | 43.6 | 206.6 | 239.3 | 243.7 | SOC2 | protein SOC2, putative |
| exon_PVP01_1434600-E1 | 235 | 1215 | 142.3 | 6.7 | 588.3 | 267.3 | 1363.9 | 1790.1 | 4364.1 | RSPH9 | radial spoke head protein 9, putative |
| exon_PVP01_1458800-E2 | 254 | 1011 | 507.2 | 518.9 | 0.0 | 107.1 | 1287.8 | 1974.9 | 1181.2 | RFC4 | replication factor C subunit 4, putative |
| exon_PVP01_1312300-E1 | 283 | 2547 | 91.3 | 180.2 | 280.6 | 170.0 | 1278.0 | 1665.8 | 150.0 | PVP01_1312300-E1 | ribonucleoside-diphosphate reductase large subunit, putative |
| exon_PVP01_1343200-E1 | 304 | 1050 | 596.2 | 741.7 | 1021.1 | 0.0 | 789.1 | 2343.0 | 818.9 | RNR | ribonucleoside-diphosphate reductase small chain, putative |
| exon_PVP01_1022500-E1 | 274 | 34386 | 19.8 | 22.4 | 72.8 | 66.1 | 25.8 | 8.3 | 20.8 | PVP01_1022500-E1 | RNA pseudouridylate synthase, putative |
| exon_PVP01_0405900-E1 | 153 | 1437 | 302.9 | 79.9 | 1492.3 | 376.7 | 411.8 | 595.5 | 698.1 | PVP01_0405900-E1 | saccharopine dehydrogenase, putative |
| exon_PVP01_1020200-E2 | 250 | 2196 | 73.3 | 74.7 | 162.7 | 295.8 | 1293.6 | 1818.4 | 783.1 | PSOP12 | secreted ookinete protein, putative |
| exon_PVP01_1453700-E3 | 359 | 1329 | 614.6 | 783.3 | 537.8 | 325.9 | 757.0 | 1448.7 | 647.0 | SHMT | serine hydroxymethyltransferase |
| exon_PVP01_0833400-E1 | 468 | 1599 | 872.5 | 979.2 | 223.5 | 474.0 | 185.1 | 512.9 | 209.1 | CCT2 | T-complex protein 1 subunit beta, putative |
| exon_PVP01_1140200-E1 | 711 | 1632 | 1457.7 | 1275.8 | 657.0 | 464.4 | 362.6 | 699.1 | 175.6 | CCT6 | T-complex protein 1 subunit zeta, putative |
| exon_PVP01_0716900-E1 | 482 | 2270 | 1034.9 | 108.3 | 157.4 | 238.5 | 338.9 | 267.0 | 463.0 | TR | telomerase RNA |
| exon_PVP01_1448300-E1 | 244 | 1098 | 733.1 | 52.3 | 1953.0 | 887.5 | 754.6 | 1623.6 | 1000.6 | TLAP1 | thioredoxin-like associated protein 1, putative |
| exon_PVP01_0916000-E1 | 221 | 1455 | 204.9 | 411.3 | 736.9 | 223.2 | 894.8 | 1274.2 | 591.0 | PVP01_0916000-E1 | vivapain-2 |

RPKM (reads per kilo base per million mapped reads) was calculated using the following equation: RPKM = (number of reads mapped to an exon * 10^3^ *10^6^) / (total number of mapped reads from a given library * exon length (in bp)). Genes shaded in light gray[^8^](#_ENREF_8) and in light yellow[^9^](#_ENREF_9) were recently reported has the 25 most expressed genes in 4 different Cambodian *P. vivax* isolates..

**Supplementary Table S12.** List of top 40 most expressed *P. vivax* *pir* transcripts from the RNA sequenced isolates.

| **Exon ID** | **Read counts** | **Transcript Length** (bp) |  | **RPKM** | | | | | | **Gene ID or Symbol** | **Product Description** |
| --- | --- | --- | --- | --- | --- | --- | --- | --- | --- | --- | --- |
|  |  |  | **62U15 (9)** | **66U15.2 (10)** | **93U15 (21)** | **93U15 (22)** | **101U15 (20)** | **101U15 (23)** | **101U15 (24)** |  |  |
| exon_PVP01_0001090-E2 | 29 | 1170 | **35.7** | **154.1** | **0.0** | **0.0** | **0.0** | **0.0** | **0.0** | PVP01_0001090 | PIR protein |
| exon_PVP01_0001090-E3 | 11 | 1170 | 20.4 | 49.0 | 0.0 | 0.0 | 0.0 | 0.0 | 0.0 | PVP01_0001090 | PIR protein |
| exon_PVP01_0002210-E2 | 19 | 2082 | 28.6 | 3.9 | 0.0 | 52.0 | 0.0 | 51.4 | 91.8 | PVP01_0002210 | PIR protein |
| exon_PVP01_0003150-E3 | 20 | 1923 | 37.2 | 8.5 | 0.0 | 0.0 | 30.8 | 74.2 | 24.8 | PVP01_0003150 | PIR protein |
| exon_PVP01_0003350-E2 | 11 | 1722 | 3.5 | 47.6 | 0.0 | 0.0 | 0.0 | 0.0 | 0.0 | PVP01_0003350 | PIR protein |
| exon_PVP01_0004400-E2 | 15 | 1044 | 11.4 | 94.2 | 0.0 | 0.0 | 0.0 | 34.2 | 0.0 | PVP01_0004400 | PIR protein |
| exon_PVP01_0004960-E1 | 19 | 1575 | 18.9 | 26.0 | 0.0 | 0.0 | 75.2 | 135.8 | 30.3 | PVP01_0004960 | PIR protein |
| exon_PVP01_0005430-E2 | 15 | 1311 | 9.1 | 56.3 | 0.0 | 0.0 | 45.1 | 27.2 | 72.9 | PVP01_0005430 | PIR protein |
| exon_PVP01_0005820-E2 | 13 | 859 | 27.8 | 57.3 | 0.0 | 0.0 | 68.9 | 41.5 | 55.6 | PVP01_0005820 | PIR protein |
| exon_PVP01_0005950-E1 | 12 | 1620 | 40.5 | 0.0 | 0.0 | 0.0 | 0.0 | 22.0 | 0.0 | PVP01_0005950 | PIR protein |
| exon_PVP01_0006650-E1 | 11 | 1113 | 10.7 | 66.3 | 0.0 | 0.0 | 0.0 | 0.0 | 0.0 | PVP01_0006650 | PIR protein |
| exon_PVP01_0009180-E2 | 29 | 1830 | 39.1 | 44.8 | 0.0 | 59.2 | 97.0 | 58.4 | 0.0 | PVP01_0009180 | PIR protein |
| exon_PVP01_0009870-E2 | 15 | 1080 | 16.6 | 68.3 | 0.0 | 100.3 | 54.8 | 33.0 | 0.0 | PVP01_0009870 | PIR protein |
| exon_PVP01_0009980-E2 | 10 | 1581 | 18.9 | 25.9 | 0.0 | 0.0 | 0.0 | 0.0 | 0.0 | PVP01_0009980 | PIR protein |
| exon_PVP01_0201500-E2 | 11 | 1476 | 24.2 | 16.7 | 0.0 | 0.0 | 0.0 | 48.3 | 0.0 | PVP01_0201500 | PIR protein |
| exon_PVP01_0201500-E3 | 88 | 1476 | 181.8 | 183.3 | 242.1 | 293.4 | 40.1 | 48.3 | 64.7 | PVP01_0201500 | PIR protein |
| exon_PVP01_0201600-E1 | 11 | 1644 | 14.5 | 29.9 | 0.0 | 0.0 | 36.0 | 0.0 | 0.0 | PVP01_0201600 | PIR protein |
| exon_PVP01_0201600-E2 | 64 | 1644 | 101.5 | 134.6 | 0.0 | 197.6 | 108.0 | 65.1 | 0.0 | PVP01_0201600 | PIR protein |
| exon_PVP01_0201600-E3 | 99 | 1644 | 97.9 | 244.3 | 0.0 | 0.0 | 252.0 | 303.6 | 58.1 | PVP01_0201600 | PIR protein |
| exon_PVP01_0201700-E2 | 59 | 1224 | 160.7 | 140.6 | 584.0 | 0.0 | 48.4 | 29.1 | 39.0 | PVP01_0201700 | PIR protein |
| exon_PVP01_0201700-E3 | 38 | 1224 | 73.1 | 133.9 | 292.0 | 0.0 | 0.0 | 29.1 | 39.0 | PVP01_0201700 | PIR protein |
| exon_PVP01_0201800-E2 | 50 | 1140 | 78.5 | 237.3 | 0.0 | 95.0 | 0.0 | 31.3 | 0.0 | PVP01_0201800 | PIR protein |
| exon_PVP01_0201900-E1 | 106 | 792 | 436.6 | 393.3 | 0.0 | 0.0 | 298.9 | 225.1 | 60.3 | PVP01_0201900 | PIR protein |
| exon_PVP01_0201900-E2 | 45 | 792 | 173.1 | 144.9 | 0.0 | 273.4 | 74.7 | 225.1 | 0.0 | PVP01_0201900 | PIR protein |
| exon_PVP01_0217400-E2 | 10 | 2187 | 8.2 | 11.2 | 0.0 | 0.0 | 27.1 | 0.0 | 65.5 | PVP01_0217400 | PIR protein |
| exon_PVP01_0403200-E2 | 10 | 1224 | 24.4 | 20.1 | 0.0 | 0.0 | 0.0 | 29.1 | 39.0 | PVP01_0403200 | PIR protein |
| exon_PVP01_0502900-E2 | 18 | 1350 | 57.4 | 18.2 | 264.7 | 0.0 | 43.8 | 0.0 | 0.0 | PVP01_0502900 | PIR protein |
| exon_PVP01_0533800-E2 | 10 | 1392 | 17.1 | 23.6 | 256.8 | 77.8 | 0.0 | 0.0 | 0.0 | PVP01_0533800 | PIR protein |
| exon_PVP01_0534100-E2 | 16 | 1155 | 0.0 | 106.5 | 309.4 | 0.0 | 0.0 | 0.0 | 0.0 | PVP01_0534100 | PIR protein |
| exon_PVP01_0624100-E2 | 10 | 1572 | 11.4 | 31.3 | 0.0 | 68.9 | 0.0 | 0.0 | 0.0 | PVP01_0624100 | PIR protein |
| exon_PVP01_0624300-E2 | 20 | 1152 | 41.4 | 71.2 | 0.0 | 0.0 | 0.0 | 61.9 | 0.0 | PVP01_0624300 | PIR protein |
| exon_PVP01_0624400-E2 | 11 | 1311 | 36.4 | 6.3 | 0.0 | 82.6 | 0.0 | 27.2 | 0.0 | PVP01_0624400 | PIR protein |
| exon_PVP01_0949400-E2 | 25 | 1260 | 66.2 | 58.6 | 0.0 | 0.0 | 47.0 | 28.3 | 0.0 | PVP01_0949400 | PIR protein |
| exon_PVP01_0949600-E2 | 15 | 1398 | 29.9 | 41.0 | 0.0 | 0.0 | 0.0 | 25.5 | 0.0 | PVP01_0949600 | PIR protein |
| exon_PVP01_0950000-E2 | 19 | 1389 | 17.2 | 35.4 | 0.0 | 77.9 | 42.6 | 51.3 | 172.0 | PVP01_0950000 | PIR protein |
| exon_PVP01_1147700-E2 | 11 | 1179 | 15.2 | 48.7 | 0.0 | 91.8 | 0.0 | 0.0 | 0.0 | PVP01_1147700 | PIR protein |
| exon_PVP01_1148600-E1 | 12 | 1815 | 0.0 | 54.2 | 0.0 | 0.0 | 0.0 | 0.0 | 0.0 | PVP01_1148600 | PIR protein |
| exon_PVP01_1272200-E2 | 14 | 1032 | 11.6 | 87.4 | 0.0 | 0.0 | 0.0 | 34.5 | 0.0 | PVP01_1272200 | PIR protein |
| exon_PVP01_1272300-E2 | 15 | 1056 | 28.2 | 69.9 | 0.0 | 102.5 | 0.0 | 0.0 | 0.0 | PVP01_1272300 | PIR protein |
| exon_PVP01_1272400-E2 | 13 | 1110 | 16.1 | 59.1 | 0.0 | 97.5 | 53.3 | 0.0 | 0.0 | PVP01_1272400 | PIR protein |
| exon_PVP01_1272400-E3 | 16 | 1110 | 37.6 | 51.7 | 0.0 | 0.0 | 53.3 | 32.1 | 0.0 | PVP01_1272400 | PIR protein |
| exon_PVP01_1272500-E2 | 26 | 1059 | 33.8 | 139.3 | 0.0 | 102.2 | 0.0 | 0.0 | 45.1 | PVP01_1272500 | PIR protein, pseudogene |
| exon_PVP01_1347800-E2 | 12 | 1359 | 35.1 | 12.1 | 263.0 | 0.0 | 0.0 | 26.2 | 0.0 | PVP01_1347800 | PIR protein |
| exon_PVP01_1470800-E2 | 13 | 1353 | 17.6 | 48.5 | 0.0 | 0.0 | 0.0 | 26.4 | 0.0 | PVP01_1470800 | PIR protein |
| exon_PVP01_1470800-E3 | 15 | 1353 | 22.0 | 54.5 | 0.0 | 80.0 | 0.0 | 0.0 | 0.0 | PVP01_1470800 | PIR protein |
| exon_PVP01_1471200-E2 | 10 | 1356 | 4.4 | 42.3 | 0.0 | 0.0 | 0.0 | 52.6 | 0.0 | PVP01_1471200 | PIR protein |
| exon_PVP01_1471200-E3 | 15 | 1356 | 30.8 | 42.3 | 0.0 | 0.0 | 0.0 | 26.3 | 0.0 | PVP01_1471200 | PIR protein |
| exon_PVP01_1471700-E2 | 15 | 1149 | 20.8 | 78.5 | 0.0 | 0.0 | 0.0 | 0.0 | 0.0 | PVP01_1471700 | PIR protein, pseudogene |

RPKM (reads per kilo base per million mapped reads) was calculated using the following equation: RPKM = (number of reads mapped to an exon * 10^3^ *10^6^) / (total number of mapped reads from a given library * exon length (in bp)). Gene shaded in light gray was recently reported as differentially expressed gene belonging to multigene families in Cambodian *P. vivax* isolates [^8^](#_ENREF_8).

**Supplementary Table S13.** List of top 100 most expressed *P. falciparum* IT genes from the RNA sequenced *P. falciparum* S20 sample.

| **Exon ID** | **Read counts** | **Transcript Length** | **RPKM** | **Gene ID or Symbol** | **Product Description** |
| --- | --- | --- | --- | --- | --- |
| **Membrane/membrane-associated and exported proteins** | | | | | |
| exon_PfIT_020012300-E2 | 287 | 2955 | 609.605 | PfIT_020012300 | serine repeat antigen 7 |
| exon_PfIT_050006200-E2 | 489 | 5154 | 595.510 | MESA | mature parasite-infected erythrocyte surface antigen |
| exon_PfIT_120033700-E1 | 392 | 2223 | 1106.804 | MSP9 | merozoite surface protein 9 |
| exon_PfIT_020012300-E4 | 449 | 2955 | 953.703 | PfIT_020012300 | serine repeat antigen 7 |
| exon_PfIT_020006800-E2 | 532 | 1905 | 1752.834 | KAHRP | knob-associated histidine-rich protein |
| exon_PfIT_140077300-E3 | 348 | 864 | 2528.074 | EXP2 | exported protein 2 |
| exon_PfIT_050036800-E1 | 1292 | 546 | 14852.314 | ETRAMP5 | early transcribed membrane protein 5 |
| exon_PfIT_110025900-E2 | 307 | 489 | 3940.522 | EXP1 | exported protein 1 |
| exon_PfIT_080023200-E3 | 196 | 789 | 1559.205 | 14-3-3I | 14-3-3 protein |
| **Kinase or kinase-like proteins** | | | | | |
| exon_PfIT_090027000-E1 | 278 | 1251 | 1394.799 | PGK | phosphoglycerate kinase |
| exon_PfIT_060030600-E3 | 185 | 1536 | 755.970 | PyrK | pyruvate kinase |
| **Conserved *Plasmodium* spp. protein, unknown function** | | | | | |
| exon_PfIT_100026000-E1 | 188 | 6801 | 173.504 | PfIT_100026000 | conserved Plasmodium protein (10b antigen), unknown function |
| exon_PfIT_070020600-E1 | 353 | 1569 | 1412.134 | PfIT_070020600 | conserved Plasmodium protein, unknown function |
| exon_PfIT_140034300-E1 | 216 | 846 | 1602.535 | PfIT_140034300 | conserved protein, unknown function |
| exon_PfIT_120042600-E2 | 189 | 633 | 1874.055 | PfIT_120042600 | conserved protein, unknown function |
| exon_PfIT_090030400-E2 | 284 | 654 | 2725.617 | PfIT_090030400 | conserved Plasmodium protein, unknown function |
| exon_PfIT_070025300-E1 | 417 | 735 | 3561.008 | PfIT_070025300 | conserved Plasmodium protein, unknown function |
| **Other general metabolic proteins and enzymes** | | | | | |
| exon_PfIT_030014400-E1 | 402 | 339 | 7443.044 | PfP2 | 60S acidic ribosomal protein P2 |
| exon_PfIT_030026800-E1 | 486 | 516 | 5911.679 | CYP19A | peptidyl-prolyl cis-trans isomerase |
| exon_PfIT_040020300-E2 | 335 | 618 | 3402.362 | PfIT_040020300 | 60S ribosomal protein L15, putative |
| exon_PfIT_040026900-E2 | 316 | 513 | 3866.286 | RPS19 | 40S ribosomal protein S19 |
| exon_PfIT_050012300-E1 | 379 | 1236 | 1924.620 | RPL4 | 60S ribosomal protein L4 |
| exon_PfIT_050016100-E1 | 201 | 516 | 2444.954 | TCTP | translationally-controlled tumor protein homolog |
| exon_PfIT_050016600-E1 | 215 | 30066 | 44.884 | PfIT_050016600 | RNA pseudouridylate synthase, putative |
| exon_PfIT_050018400-E1 | 406 | 738 | 3452.979 | PNP | purine nucleoside phosphorylase |
| exon_PfIT_050022100-E2 | 513 | 783 | 4112.253 | RPL2 | 60S ribosomal protein L2 |
| exon_PfIT_050022200-E2 | 230 | 498 | 2898.830 | PfIT_050022200 | 60S ribosomal protein L12, putative |
| exon_PfIT_050024600-E1 | 338 | 402 | 5277.338 | RPS24 | 40S ribosomal protein S24 |
| exon_PfIT_050025200-E3 | 363 | 570 | 3997.201 | PfIT_050025200 | 40S ribosomal protein S9, putative |
| exon_PfIT_050026100-E1 | 260 | 1440 | 1133.274 | SAHH | S-adenosyl-L-homocysteine hydrolase |
| exon_PfIT_050036700:rRNA-E1 | 3781 | 3925 | 6046.322 | PfIT_050036700:rRNA | rRNA |
| exon_PfIT_060012900-E1 | 909 | 1245 | 4582.672 | OAT | ornithine aminotransferase |
| exon_PfIT_060018500-E3 | 200 | 549 | 2286.556 | RPL19 | 60S ribosomal protein L19 |
| exon_PfIT_060021600-E1 | 5490 | 399 | 86362.200 | H2A | histone H2A |
| exon_PfIT_060021700-E1 | 848 | 411 | 12950.254 | H3.3 | histone H3 variant, putative |
| exon_PfIT_060022100-E2 | 206 | 447 | 2892.570 | PfIT_060022100 | 60S ribosomal protein L27a, putative |
| exon_PfIT_070013300-E1 | 661 | 2238 | 1853.812 | HSP90 | heat shock protein 90 |
| exon_PfIT_070018300-E1 | 280 | 372 | 4724.320 | H2B.Z | histone H2B variant |
| exon_PfIT_070023800-E2 | 293 | 522 | 3523.071 | PfIT_070023800 | 60S ribosomal protein L11a, putative |
| exon_PfIT_070025800-E2 | 190 | 588 | 2028.152 | PfIT_070025800 | 40S ribosomal protein S5, putative |
| exon_PfIT_070029800:rRNA-E1 | 1235 | 3922 | 1976.440 | PfIT_070029800:rRNA | rRNA |
| exon_PfIT_080007300-E1 | 226 | 4194 | 338.224 | GDH3 | glutamate dehydrogenase, putative |
| exon_PfIT_080019200-E1 | 444 | 747 | 3730.668 | ALBA1 | DNA/RNA-binding protein Alba 1 |
| exon_PfIT_080023900-E1 | 956 | 2046 | 2932.760 | PfIT_080023900 | heat shock protein 70 |
| exon_PfIT_080031700-E2 | 591 | 972 | 3816.326 | RACK | receptor for activated c kinase |
| exon_PfIT_080032800-E2 | 364 | 1452 | 1573.472 | PDI8 | protein disulfide isomerase |
| exon_PfIT_080036400-E1 | 274 | 2040 | 843.033 | PfIT_080036400 | heat shock protein 70 |
| exon_PfIT_090022500-E2 | 1051 | 1959 | 3367.383 | PfIT_090022500 | heat shock protein 70 |
| exon_PfIT_090026700-E1 | 358 | 1209 | 1858.579 | SAMS | S-adenosylmethionine synthetase |
| exon_PfIT_100005400-E1 | 338 | 273 | 7771.025 | ACBP2 | acyl-CoA binding protein, isoform 2, ACBP2 |
| exon_PfIT_100012700-E2 | 241 | 1338 | 1130.538 | PfIT_100012700 | tubulin beta chain |
| exon_PfIT_100016400-E3 | 730 | 696 | 6583.212 | HGPRT | hypoxanthine-guanine phosphoribosyltransferase |
| exon_PfIT_100019900-E2 | 458 | 1341 | 2143.685 | ENO | enolase |
| exon_PfIT_100020400-E2 | 299 | 2475 | 758.264 | GBP | glycophorin binding protein |
| exon_PfIT_100028000-E1 | 265 | 9954 | 167.098 | CHD1 | chromodomain-helicase-DNA-binding protein 1 homolog, putative |
| exon_PfIT_100030800-E2 | 269 | 792 | 2131.824 | RPS2 | 40S ribosomal protein S2 |
| exon_PfIT_100037000-E1 | 228 | 4260 | 335.931 | AdoMetDC/ODC | S-adenosylmethionine decarboxylase/ornithine decarboxylase |
| exon_PfIT_100041000-E1 | 196 | 906 | 1357.851 | ADT | ADP/ATP transporter on adenylate translocase |
| exon_PfIT_110007600-E1 | 193 | 357 | 3393.230 | RPP1 | 60S acidic ribosomal protein P1, putative |
| exon_PfIT_110009500-E1 | 1690 | 312 | 33998.234 | H4 | histone H4 |
| exon_PfIT_110009600-E1 | 2048 | 354 | 36312.064 | H2B | histone H2B |
| exon_PfIT_110010000-E2 | 227 | 786 | 1812.707 | PfIT_110010000 | 40S ribosomal protein S4, putative |
| exon_PfIT_110013200-E1 | 192 | 1032 | 1167.739 | ERC | endoplasmic reticulum-resident calcium binding protein |
| exon_PfIT_110021000-E1 | 410 | 2103 | 1223.683 | DPAP1 | dipeptidyl aminopeptidase 1 |
| exon_PfIT_110022000-E2 | 341 | 645 | 3318.325 | RAN | GTP-binding nuclear protein RAN/TC4 |
| exon_PfIT_110024400-E3 | 286 | 753 | 2383.940 | PGM1 | phosphoglycerate mutase, putative |
| exon_PfIT_110034600-E1 | 259 | 264 | 6157.722 | RPL38 | 60S ribosomal protein L38 |
| exon_PfIT_110034700-E1 | 885 | 951 | 5840.997 | PfP0 | 60S ribosomal protein P0 |
| exon_PfIT_120008500-E1 | 252 | 294 | 5379.940 | HMGB1 | high mobility group protein B1 |
| exon_PfIT_120009300-E3 | 237 | 1044 | 1424.860 | NAPL | nucleosome assembly protein |
| exon_PfIT_120009800-E1 | 558 | 486 | 7206.463 | EIF5A | eukaryotic translation initiation factor 5A |
| exon_PfIT_120018100-E1 | 194 | 4134 | 294.548 | EIF3A | eukaryotic translation initiation factor 3 subunit A, putative |
| exon_PfIT_120027400-E1 | 254 | 2466 | 646.495 | GRP94 | endoplasmin, putative |
| exon_PfIT_120034400-E2 | 194 | 351 | 3469.116 | MIF | macrophage migration inhibitory factor |
| exon_PfIT_120051400-E1 | 216 | 1131 | 1198.714 | ACT1 | actin I |
| exon_PfIT_130007900-E1 | 203 | 585 | 2178.033 | PfIT_130007900 | 40S ribosomal protein S7, putative |
| exon_PfIT_130017000-E1 | 274 | 3258 | 527.866 | M1AAP | M1-family alanyl aminopeptidase |
| exon_PfIT_130028400-E2 | 213 | 573 | 2333.185 | null | 60S ribosomal protein L6, putative |
| exon_PfIT_130030200-E1 | 1418 | 951 | 9358.796 | LDH | L-lactate dehydrogenase |
| exon_PfIT_130043600-E2 | 396 | 1236 | 2010.949 | PfIT_130043600 | elongation factor 1-gamma, putative |
| exon_PfIT_130047300-E1 | 375 | 921 | 2555.618 | PfIT_130047300 | 40S ribosomal protein S6 |
| exon_PfIT_130048300-E4 | 320 | 801 | 2507.504 | PMT | phosphoethanolamine N-methyltransferase |
| exon_PfIT_130052500-E1 | 500 | 1269 | 2473.048 | NT1 | nucleoside transporter 1 |
| exon_PfIT_130057800-E2 | 202 | 627 | 2022.125 | PfIT_130057800 | thioredoxin-related protein, putative |
| exon_PfIT_130064200-E1 | 413 | 456 | 5684.725 | RPS15 | 40S ribosomal protein S15 |
| exon_PfIT_130067200-E1 | 251 | 825 | 1909.607 | PCNA1 | proliferating cell nuclear antigen 1 |
| exon_PfIT_140014700-E1 | 201 | 1356 | 930.381 | HAP | plasmepsin III |
| exon_PfIT_140015200-E1 | 287 | 657 | 2741.832 | PfIT_140015200 | 40S ribosomal protein S8e, putative |
| exon_PfIT_140020900-E1 | 504 | 660 | 4793.038 | PfIT_140020900 | 60S ribosomal protein L10, putative |
| exon_PfIT_140030600-E2 | 255 | 885 | 1808.511 | PfIT_140030600 | 60S ribosomal protein L5, putative |
| exon_PfIT_140032400-E1 | 270 | 486 | 3486.998 | RPL21 | 60S ribosomal protein L21 |
| exon_PfIT_140045400-E1 | 270 | 588 | 2882.111 | Trx-Px1 | thioredoxin peroxidase 1 |
| exon_PfIT_140046400-E2 | 281 | 747 | 2361.076 | TIM | triosephosphate isomerase |
| exon_PfIT_140051200-E2 | 777 | 1110 | 4393.618 | FBPA | fructose-bisphosphate aldolase |
| exon_PfIT_140057500-E1 | 378 | 2499 | 949.401 | eEF2 | elongation factor 2 |
| exon_PfIT_140060100-E2 | 221 | 828 | 1675.275 | P23 | co-chaperone p23 |
| exon_PfIT_140060800-E1 | 190 | 2328 | 512.265 | APP | aminopeptidase P |
| exon_PfIT_140067700-E1 | 208 | 204 | 6399.668 | PfIT_140067700 | 40S ribosomal protein S28e, putative |
| exon_PfIT_140069200-E2 | 3131 | 1014 | 19380.696 | GAPDH | glyceraldehyde-3-phosphate dehydrogenase |
| exon_PfIT_140072200-E3 | 237 | 666 | 2233.564 | PfIT_140072200 | 40S ribosomal protein S3 |

RPKM (reads per kilo base per million mapped reads) was calculated using the following equation: RPKM = (number of reads mapped to an exon * 10^3^ *10^6^) / (total number of mapped reads from a given library * exon length (in bp)).

**Supplementary Table S14.** Patient’s expression profile: list of top 100 most expressed human genes from the RNA sequenced field isolate.

| **Exon ID** | **Read counts** | **Transcript Length** |  | **RPKM** | | | | | | **Gene ID or Symbol** | **Product Description** |
| --- | --- | --- | --- | --- | --- | --- | --- | --- | --- | --- | --- |
|  |  |  | **62U15 (9)** | **66U15.2 (10)** | **93U15 (21)** | **93U15 (22)** | **101U15 (20)** | **101U15 (23)** | **101U15 (24)** |  |  |
| exon_ENSG00000165527.6-E2 | 71 | 3865 | 241.6 | 536.2 | 6536.4 | 628.8 | 803.5 | 3711.1 | 996.1 | ARF6 | ADP ribosylation factor 6 |
| exon_ENSG00000169877.9-E7 | 21 | 524 | 0.0 | 659.2 | 0.0 | 0.0 | 0.0 | 2566.2 | 31225.0 | AHSP | alpha hemoglobin stabilizing protein |
| exon_ENSG00000183684.7-E13 | 22 | 1088 | 858.2 | 0.0 | 7739.9 | 2233.6 | 0.0 | 2883.8 | 3538.5 | ALYREF | Aly/REF export factor |
| exon_ENSG00000111875.7-E6 | 10 | 2362 | 0.0 | 146.2 | 445.7 | 0.0 | 0.0 | 569.3 | 2037.4 | ASF1A | anti-silencing function 1A histone chaperone |
| exon_ENSG00000136888.6-E5 | 11 | 1080 | 864.5 | 959.5 | 0.0 | 1125.1 | 0.0 | 2490.2 | 0.0 | ATP6V1G1 | ATPase, H+ transporting, lysosomal 13kDa, V1 subunit G1 |
| exon_ENSG00000133639.4-E4 | 35 | 4680 | 997.6 | 738.1 | 1124.6 | 519.3 | 663.6 | 957.8 | 205.7 | BTG1 | B-cell translocation gene 1, anti-proliferative |
| exon_ENSG00000171552.12-E15 | 10 | 2610 | 357.7 | 132.3 | 0.0 | 0.0 | 1189.9 | 858.7 | 368.8 | BCL2L1 | BCL2-like 1 |
| exon_ENSG00000221869.4-E1 | 16 | 2178 | 857.4 | 0.0 | 2416.5 | 557.9 | 0.0 | 1234.8 | 883.8 | CEBPD | CCAAT/enhancer binding protein (C/EBP), delta |
| exon_ENSG00000026508.16-E94 | 11 | 4589 | 0.0 | 526.9 | 0.0 | 264.8 | 0.0 | 195.3 | 209.7 | CD44 | CD44 molecule (Indian blood group) |
| exon_ENSG00000110848.8-E8 | 13 | 1676 | 1114.2 | 206.1 | 0.0 | 1450.0 | 2779.5 | 802.3 | 1148.5 | CD69 | CD69 molecule |
| exon_ENSG00000123975.4-E3 | 28 | 613 | 0.0 | 0.0 | 15454.6 | 3964.3 | 5066.2 | 4387.2 | 14130.8 | CKS2 | CDC28 protein kinase regulatory subunit 2 |
| exon_ENSG00000123975.4-E2 | 16 | 613 | 0.0 | 0.0 | 13737.4 | 1982.2 | 0.0 | 3656.0 | 3140.2 | CKS2 | CDC28 protein kinase regulatory subunit 2 |
| exon_ENSG00000237350.1-E1 | 37 | 576 | 1621.0 | 599.7 | 23757.3 | 6328.5 | 8087.5 | 7781.8 | 10025.7 | CDC42P6 | cell division cycle 42 pseudogene 6 |
| exon_ENSG00000243317.7-E7 | 10 | 2450 | 0.0 | 282.0 | 0.0 | 0.0 | 633.8 | 365.9 | 1964.2 | C7orf73 | chromosome 7 open reading frame 73 |
| exon_ENSG00000160124.9-E4 | 10 | 769 | 0.0 | 0.0 | 2737.7 | 1580.1 | 0.0 | 4080.1 | 0.0 | CCDC58 | coiled-coil domain containing 58 |
| exon_ENSG00000120129.5-E4 | 10 | 2019 | 1387.4 | 171.1 | 521.4 | 0.0 | 769.1 | 888.0 | 0.0 | DUSP1 | dual specificity phosphatase 1 |
| exon_ENSG00000264364.2-E3 | 11 | 6805 | 0.0 | 0.0 | 618.7 | 178.6 | 0.0 | 197.6 | 424.3 | DYNLL2 | dynein, light chain, LC8-type 2 |
| exon_ENSG00000115380.19-E34 | 14 | 3024 | 0.0 | 0.0 | 3480.9 | 401.8 | 0.0 | 444.7 | 0.0 | EFEMP1 | EGF containing fibulin-like extracellular matrix protein 1 |
| exon_ENSG00000066044.13-E15 | 11 | 2312 | 0.0 | 0.0 | 1365.9 | 0.0 | 671.6 | 1163.2 | 416.3 | ELAVL1 | ELAV like RNA binding protein 1 |
| exon_ENSG00000196205.8-E1 | 16 | 1389 | 1344.4 | 1740.8 | 0.0 | 874.8 | 2235.8 | 1290.8 | 0.0 | EEF1A1P5 | eukaryotic translation elongation factor 1 alpha 1 pseudogene |
| exon_ENSG00000128692.8-E1 | 13 | 984 | 948.9 | 0.0 | 6418.5 | 1234.8 | 0.0 | 1822.1 | 978.1 | EIF2S2P4 | eukaryotic translation initiation factor 2 subunit 2 beta pseudogene 4 |
| exon_ENSG00000253626.3-E1 | 389 | 3840 | 243.2 | 0.0 | 46600.9 | 5062.8 | 5256.9 | 19493.3 | 5514.1 | EIF5AL1 | eukaryotic translation initiation factor 5A-like 1 |
| exon_ENSG00000048828.16-E26 | 10 | 5118 | 0.0 | 0.0 | 411.3 | 237.4 | 303.4 | 525.5 | 0.0 | FAM120A | family with sequence similarity 120A |
| exon_ENSG00000124098.9-E5 | 11 | 3046 | 0.0 | 793.8 | 0.0 | 0.0 | 509.8 | 441.5 | 0.0 | FAM210B | family with sequence similarity 210 member B |
| exon_ENSG00000183508.4-E2 | 19 | 5751 | 487.1 | 60.1 | 0.0 | 211.3 | 1350.0 | 467.6 | 502.1 | FAM46C | family with sequence similarity 46 member C |
| exon_ENSG00000087086.13-E3 | 13 | 878 | 0.0 | 3934.2 | 0.0 | 1383.9 | 0.0 | 510.5 | 1096.2 | FTL | ferritin, light polypeptide |
| exon_ENSG00000087086.13-E4 | 10 | 772 | 0.0 | 3132.1 | 0.0 | 0.0 | 0.0 | 580.6 | 2493.4 | FTL | ferritin, light polypeptide |
| exon_ENSG00000226608.3-E1 | 61 | 528 | 5305.2 | 31402.1 | 0.0 | 0.0 | 0.0 | 4244.6 | 9114.2 | FTLP3 | ferritin, light polypeptide pseudogene 3 |
| exon_ENSG00000169508.6-E2 | 12 | 1700 | 2197.0 | 1219.1 | 0.0 | 0.0 | 0.0 | 527.3 | 0.0 | GPR183 | G protein-coupled receptor 183 |
| exon_ENSG00000179144.4-E2 | 21 | 1256 | 4460.4 | 2750.2 | 838.1 | 967.4 | 0.0 | 713.7 | 766.3 | GIMAP7 | GTPase, IMAP family member 7 |
| exon_ENSG00000132475.8-E29 | 11 | 2787 | 335.0 | 123.9 | 0.0 | 0.0 | 0.0 | 1125.8 | 690.7 | H3F3B | H3 histone, family 3B (H3.3B) |
| exon_ENSG00000044574.7-E8 | 17 | 3908 | 716.8 | 441.9 | 269.4 | 0.0 | 397.3 | 573.5 | 492.6 | HSPA5 | heat shock protein family A (Hsp70) member 5 |
| exon_ENSG00000206177.6-E4 | 12 | 592 | 0.0 | 583.5 | 0.0 | 0.0 | 0.0 | 757.1 | 16257.8 | HBM | hemoglobin subunit mu |
| exon_ENSG00000197451.10-E23 | 11 | 1796 | 0.0 | 0.0 | 2344.4 | 0.0 | 864.6 | 1247.9 | 535.9 | HNRNPAB | heterogeneous nuclear ribonucleoprotein A/B |
| exon_ENSG00000152795.17-E15 | 10 | 4139 | 0.0 | 0.0 | 1271.6 | 0.0 | 0.0 | 216.6 | 697.6 | HNRNPDL | heterogeneous nuclear ribonucleoprotein D like |
| exon_ENSG00000211895.4-E2 | 23 | 1022 | 4568.0 | 676.0 | 0.0 | 1188.9 | 6077.5 | 3508.6 | 2825.2 | IGHA1 | immunoglobulin heavy constant alpha 1 |
| exon_ENSG00000211895.4-E3 | 25 | 1112 | 1679.3 | 931.9 | 1893.2 | 2185.4 | 1396.4 | 4433.9 | 3462.1 | IGHA1 | immunoglobulin heavy constant alpha 1 |
| exon_ENSG00000211895.4-E1 | 17 | 2619 | 1426.1 | 395.7 | 0.0 | 463.9 | 0.0 | 1026.9 | 1102.5 | IGHA1 | immunoglobulin heavy constant alpha 1 |
| exon_ENSG00000211896.7-E1 | 11 | 1128 | 1655.5 | 306.2 | 0.0 | 5385.9 | 0.0 | 1192.1 | 0.0 | IGHG1 | immunoglobulin heavy constant gamma 1 (G1m marker) |
| exon_ENSG00000211899.8-E4 | 17 | 1485 | 6916.3 | 0.0 | 0.0 | 818.2 | 0.0 | 1509.2 | 0.0 | IGHM | immunoglobulin heavy constant mu |
| exon_ENSG00000211899.8-E3 | 11 | 1485 | 4401.3 | 232.6 | 0.0 | 818.2 | 1045.7 | 301.8 | 0.0 | IGHM | immunoglobulin heavy constant mu |
| exon_ENSG00000211677.2-E1 | 83 | 462 | 82861.4 | 4486.0 | 0.0 | 52600.3 | 6722.1 | 7761.5 | 12499.5 | IGLC2 | immunoglobulin lambda constant 2 (Kern-Oz- marker) |
| exon_ENSG00000211679.2-E1 | 71 | 462 | 38399.2 | 0.0 | 0.0 | 42080.2 | 10083.1 | 16493.3 | 33332.1 | IGLC3 | immunoglobulin lambda constant 3 (Kern-Oz+ marker) |
| exon_ENSG00000171223.5-E1 | 21 | 1820 | 1539.1 | 759.2 | 3470.2 | 1335.2 | 0.0 | 1477.7 | 0.0 | JUNB | jun B proto-oncogene |
| exon_ENSG00000130522.5-E3 | 13 | 1863 | 1002.4 | 741.6 | 0.0 | 1304.4 | 833.5 | 721.8 | 516.6 | JUND | jun D proto-oncogene |
| exon_ENSG00000104660.17-E14 | 12 | 3173 | 882.8 | 762.0 | 0.0 | 765.9 | 0.0 | 0.0 | 0.0 | LEPROTL1 | leptin receptor overlapping transcript-like 1 |
| exon_ENSG00000227036.6-E4 | 13 | 2265 | 0.0 | 0.0 | 464.7 | 536.5 | 685.6 | 0.0 | 4249.3 | LINC00511 | long intergenic non-protein coding RNA 511 |
| exon_ENSG00000232024.2-E1 | 12 | 588 | 0.0 | 0.0 | 5370.6 | 2066.4 | 2640.8 | 5336.1 | 0.0 | LSM12P1 | LSM12 pseudogene 1 |
| exon_ENSG00000162511.7-E12 | 16 | 2223 | 2520.1 | 776.9 | 0.0 | 0.0 | 1397.0 | 403.3 | 433.0 | LAPTM5 | lysosomal protein transmembrane 5 |
| exon_ENSG00000206503.11-E22 | 32 | 1868 | 2999.1 | 1849.2 | 0.0 | 1300.9 | 1662.5 | 1439.7 | 3091.4 | HLA-A | major histocompatibility complex, class I, A |
| exon_ENSG00000234745.9-E19 | 37 | 1547 | 4828.5 | 893.1 | 0.0 | 2356.3 | 8030.0 | 2607.7 | 3110.7 | HLA-B | major histocompatibility complex, class I, B |
| exon_ENSG00000204525.15-E22 | 36 | 1880 | 1986.6 | 1837.4 | 559.9 | 1292.6 | 3303.8 | 1907.4 | 3583.6 | HLA-C | major histocompatibility complex, class I, C |
| exon_ENSG00000204592.8-E12 | 58 | 2601 | 1076.9 | 4780.9 | 0.0 | 934.3 | 1791.0 | 1723.3 | 1480.1 | HLA-E | major histocompatibility complex, class I, E |
| exon_ENSG00000162840.4-E1 | 17 | 183 | 15306.7 | 3775.1 | 0.0 | 0.0 | 0.0 | 4898.7 | 52593.7 | MT2P1 | metallothionein 2 pseudogene 1 |
| exon_ENSG00000211459.2-E1 | 39 | 954 | 0.0 | 12310.7 | 0.0 | 3821.0 | 1627.7 | 469.8 | 0.0 | MT-RNR1 | mitochondrially encoded 12S RNA |
| exon_ENSG00000210082.2-E1 | 442 | 1559 | 13775.0 | 71787.7 | 2025.6 | 17925.9 | 17928.4 | 6037.7 | 18520.8 | MT-RNR2 | mitochondrially encoded 16S RNA |
| exon_ENSG00000198899.2-E1 | 22 | 681 | 2742.2 | 6594.0 | 0.0 | 3568.5 | 2280.2 | 1316.4 | 2826.6 | MT-ATP6 | mitochondrially encoded ATP synthase 6 |
| exon_ENSG00000198727.2-E1 | 28 | 1141 | 818.3 | 3330.1 | 0.0 | 8519.3 | 0.0 | 2357.0 | 1687.1 | MT-CYB | mitochondrially encoded cytochrome b |
| exon_ENSG00000198804.2-E1 | 78 | 1542 | 6660.7 | 9856.4 | 0.0 | 3151.9 | 1007.0 | 3778.8 | 3120.8 | MT-CO1 | mitochondrially encoded cytochrome c oxidase I |
| exon_ENSG00000198712.1-E1 | 59 | 684 | 10920.5 | 8585.1 | 1538.9 | 14211.3 | 6810.5 | 5897.8 | 18292.4 | MT-CO2 | mitochondrially encoded cytochrome c oxidase II |
| exon_ENSG00000198938.2-E1 | 47 | 784 | 2381.9 | 7049.5 | 0.0 | 15498.3 | 3961.2 | 2286.9 | 15959.2 | MT-CO3 | mitochondrially encoded cytochrome c oxidase III |
| exon_ENSG00000198888.2-E1 | 60 | 956 | 976.7 | 18427.4 | 0.0 | 5084.0 | 1624.3 | 937.7 | 1006.8 | MT-ND1 | mitochondrially encoded NADH:ubiquinone oxidoreductase core subunit 1 |
| exon_ENSG00000198763.3-E1 | 32 | 1042 | 0.0 | 6630.0 | 1010.2 | 5830.5 | 1490.2 | 860.3 | 2771.0 | MT-ND2 | mitochondrially encoded NADH:ubiquinone oxidoreductase core subunit 2 |
| exon_ENSG00000198886.2-E1 | 62 | 1378 | 1355.2 | 8272.1 | 0.0 | 7935.9 | 5634.2 | 1301.1 | 6286.0 | MT-ND4 | mitochondrially encoded NADH:ubiquinone oxidoreductase core subunit 4 |
| exon_ENSG00000198786.2-E1 | 19 | 1812 | 515.3 | 1906.3 | 0.0 | 0.0 | 2570.9 | 247.4 | 2124.6 | MT-ND5 | mitochondrially encoded NADH:ubiquinone oxidoreductase core subunit 5 |
| exon_ENSG00000147065.16-E23 | 14 | 3944 | 473.5 | 350.3 | 0.0 | 616.2 | 787.4 | 454.6 | 0.0 | MSN | moesin |
| exon_ENSG00000118680.12-E7 | 10 | 1210 | 2315.0 | 856.4 | 0.0 | 0.0 | 0.0 | 370.4 | 2386.3 | MYL12B | myosin light chain 12B |
| exon_ENSG00000198618.5-E1 | 10 | 498 | 3749.8 | 1387.2 | 2113.7 | 2439.9 | 6236.1 | 0.0 | 3865.3 | PPIAP22 | peptidylprolyl isomerase A (cyclophilin A) pseudogene 22 |
| exon_ENSG00000176407.17-E11 | 14 | 7568 | 0.0 | 45.6 | 278.2 | 0.0 | 0.0 | 533.0 | 254.4 | KCMF1 | potassium channel modulatory factor 1 |
| exon_ENSG00000115233.11-E11 | 10 | 4890 | 0.0 | 0.0 | 645.8 | 0.0 | 317.5 | 550.0 | 0.0 | PSMD14 | proteasome 26S subunit, non-ATPase 14 |
| exon_ENSG00000221823.10-E10 | 16 | 3023 | 0.0 | 0.0 | 1392.8 | 0.0 | 0.0 | 1779.3 | 0.0 | PPP3R1 | protein phosphatase 3 regulatory subunit B, alpha |
| exon_ENSG00000197744.5-E1 | 19 | 327 | 2855.4 | 6338.0 | 0.0 | 14863.2 | 4748.6 | 5482.9 | 8829.9 | PTMAP2 | prothymosin, alpha pseudogene 2 |
| exon_ENSG00000067560.10-E18 | 17 | 2031 | 459.7 | 510.2 | 1554.8 | 598.3 | 764.5 | 1544.9 | 473.9 | RHOA | ras homolog family member A |
| exon_ENSG00000236552.2-E1 | 13 | 612 | 3051.3 | 564.4 | 0.0 | 3970.8 | 0.0 | 1464.8 | 9435.9 | RPL13AP5 | ribosomal protein L13a pseudogene 5 |
| exon_ENSG00000213442.5-E1 | 33 | 528 | 7073.5 | 7850.5 | 0.0 | 11506.3 | 2940.9 | 848.9 | 18228.5 | RPL18AP3 | ribosomal protein L18a pseudogene 3 |
| exon_ENSG00000165502.6-E2 | 14 | 746 | 1251.6 | 1852.1 | 1411.0 | 1628.8 | 0.0 | 600.8 | 7741.0 | RPL36AL | ribosomal protein L36a like |
| exon_ENSG00000213553.4-E1 | 12 | 954 | 1957.5 | 0.0 | 2206.8 | 0.0 | 1627.7 | 2349.2 | 2017.7 | RPLP0P6 | ribosomal protein, large, P0 pseudogene 6 |
| exon_ENSG00000132819.16-E13 | 24 | 2382 | 784.0 | 725.1 | 0.0 | 0.0 | 3259.4 | 2069.9 | 404.1 | RBM38 | RNA binding motif protein 38 |
| exon_ENSG00000188404.8-E18 | 11 | 2436 | 1149.9 | 709.0 | 0.0 | 0.0 | 637.4 | 184.0 | 395.1 | SELL | selectin L |
| exon_ENSG00000167978.16-E9 | 36 | 9353 | 0.0 | 36.9 | 1463.1 | 129.9 | 0.0 | 670.9 | 720.3 | SRRM2 | serine/arginine repetitive matrix 2 |
| exon_ENSG00000124783.12-E30 | 10 | 9669 | 0.0 | 35.7 | 217.7 | 0.0 | 160.6 | 0.0 | 597.2 | SSR1 | signal sequence receptor, alpha |
| exon_ENSG00000235082.2-E1 | 10 | 304 | 0.0 | 1136.3 | 10387.8 | 3996.9 | 0.0 | 7372.2 | 0.0 | SUMO1P3 | SUMO1 pseudogene 3 (functional) |
| exon_ENSG00000211772.9-E1 | 13 | 758 | 1231.8 | 3189.9 | 0.0 | 1603.0 | 2048.5 | 1182.7 | 1269.7 | TRBC2 | T cell receptor beta constant 2 |
| exon_ENSG00000265972.5-E15 | 13 | 1509 | 0.0 | 2289.1 | 0.0 | 0.0 | 2058.0 | 0.0 | 637.8 | TXNIP | thioredoxin interacting protein |
| exon_ENSG00000034510.5-E3 | 73 | 496 | 15059.8 | 4178.5 | 8489.0 | 14698.4 | 12522.5 | 10844.3 | 64034.9 | TMSB10 | thymosin beta 10 |
| exon_ENSG00000034510.5-E2 | 10 | 496 | 1882.5 | 0.0 | 4244.5 | 2449.7 | 0.0 | 1807.4 | 7761.8 | TMSB10 | thymosin beta 10 |
| exon_ENSG00000187653.11-E1 | 140 | 135 | 110661.5 | 33263.0 | 171540.0 | 90005.0 | 23004.4 | 89645.9 | 356468.1 | TMSB4XP8 | thymosin beta 4, X-linked pseudogene 8 |
| exon_ENSG00000028137.16-E14 | 10 | 3683 | 507.0 | 375.2 | 0.0 | 329.9 | 421.6 | 0.0 | 522.7 | TNFRSF1B | tumor necrosis factor receptor superfamily member 1B |
| exon_ENSG00000225022.1-E1 | 37 | 444 | 0.0 | 0.0 | 33191.1 | 8209.9 | 3497.3 | 17161.9 | 4335.4 | UBE2D3P1 | ubiquitin conjugating enzyme E2D 3 pseudogene 1 |
| exon_ENSG00000228305.2-E1 | 38 | 654 | 0.0 | 0.0 | 28971.5 | 1857.9 | 2374.3 | 8224.4 | 8829.9 | AC016734.2 | unspecified product |
| exon_ENSG00000218175.2-E1 | 13 | 345 | 8119.2 | 4004.9 | 0.0 | 7043.9 | 4500.9 | 1299.2 | 5579.5 | AC016739.2 | unspecified product |
| exon_ENSG00000277048.1-E1 | 42 | 57 | 32761.6 | 18180.2 | 0.0 | 149218.7 | 681050.5 | 0.0 | 84426.7 | AL353644.10 | unspecified product |
| exon_ENSG00000185641.6-E1 | 18 | 373 | 0.0 | 6482.5 | 0.0 | 9772.7 | 8326.0 | 0.0 | 15482.0 | CTD-2287O16.1 | unspecified product |
| exon_ENSG00000242299.1-E1 | 17 | 459 | 2034.2 | 2257.7 | 0.0 | 7941.6 | 0.0 | 2929.6 | 14678.1 | RP11-234A1.1 | unspecified product |
| exon_ENSG00000283041.1-E1 | 14 | 1314 | 2131.8 | 262.9 | 0.0 | 2774.1 | 1181.7 | 1364.5 | 1464.9 | RP11-371A22.1 | unspecified product |
| exon_ENSG00000220842.6-E1 | 12 | 483 | 1933.1 | 5006.1 | 0.0 | 0.0 | 3214.9 | 928.0 | 3985.4 | RP11-572P18.1 | unspecified product |
| exon_ENSG00000234287.1-E1 | 14 | 255 | 0.0 | 4063.8 | 0.0 | 4765.0 | 12178.8 | 1757.8 | 26420.6 | RP11-761N21.2 | unspecified product |
| exon_ENSG00000228532.4-E1 | 22 | 288 | 0.0 | 0.0 | 40204.7 | 12656.9 | 5391.6 | 9338.1 | 3341.9 | RP1-241P17.4 | unspecified product |
| exon_ENSG00000101558.13-E23 | 19 | 6815 | 0.0 | 0.0 | 772.3 | 178.3 | 0.0 | 591.9 | 564.9 | VAPA | VAMP associated protein A |
| exon_ENSG00000152518.6-E6 | 30 | 3696 | 252.6 | 1962.6 | 284.8 | 328.8 | 420.1 | 363.8 | 520.8 | ZFP36L2 | ZFP36 ring finger protein-like 2 |

RPKM (reads per kilo base per million mapped reads) was calculated using the following equation: RPKM = (number of reads mapped to an exon * 10^3^ *10^6^) / (total number of mapped reads from a given library * exon length (in bp)).

**References:**

1 Chomczynski, P. & Sacchi, N. Single-step method of RNA isolation by acid guanidinium thiocyanate-phenol-chloroform extraction. *Anal Biochem* **162**, 156-159, doi:10.1006/abio.1987.9999 (1987).

2 Chomczynski, P. & Sacchi, N. The single-step method of RNA isolation by acid guanidinium thiocyanate-phenol-chloroform extraction: twenty-something years on. *Nat Protoc* **1**, 581-585, doi:10.1038/nprot.2006.83 (2006).

3 Kirsten Moll, I. L., Hedvig Perlmann, Artur Scherf, Mats Wahlgren. Methods in Malaria Research. *Malaria Research and Reference Reagent Resource Center (MR4) and American Type Culture Collection 10801 University Boulevard, Manassas, VA 20110-2209* (2008).

4 Segurado, A. A., di Santi, S. M. & Shiroma, M. In vivo and in vitro Plasmodium falciparum resistance to chloroquine, amodiaquine and quinine in the Brazilian Amazon. *Revista do Instituto de Medicina Tropical de Sao Paulo* **39**, 85-90, doi:10.1590/s0036-46651997000200004 (1997).

5 Auburn, S. *et al.* A new Plasmodium vivax reference sequence with improved assembly of the subtelomeres reveals an abundance of pir genes. *Wellcome Open Res* **1**, 4, doi:10.12688/wellcomeopenres.9876.1 (2016).

6 Landis, J. R. & Koch, G. G. The measurement of observer agreement for categorical data. *Biometrics* **33**, 159-174 (1977).

7 McIntyre, L. M. *et al.* RNA-seq: technical variability and sampling. *BMC Genomics* **12**, 293, doi:10.1186/1471-2164-12-293 (2011).

8 Siegel, S. V. *et al.* Analysis of Plasmodium vivax schizont transcriptomes from field isolates reveals heterogeneity of expression of genes involved in host-parasite interactions. *Sci Rep* **10**, 16667, doi:10.1038/s41598-020-73562-7 (2020).

9 Kim, A. *et al.* Characterization of P. vivax blood stage transcriptomes from field isolates reveals similarities among infections and complex gene isoforms. *Sci Rep* **7**, 7761, doi:10.1038/s41598-017-07275-9 (2017).
